# INDUCER OF CBF EXPRESSION 1 is a male fertility regulator impacting anther dehydration in *Arabidopsis*

**DOI:** 10.1371/journal.pgen.1007695

**Published:** 2018-10-04

**Authors:** Donghui Wei, Mingjia Liu, Hu Chen, Ye Zheng, Yuxiao Liu, Xi Wang, Shuhua Yang, Mingqi Zhou, Juan Lin

**Affiliations:** 1 State Key Laboratory of Genetic Engineering, Institute of Plant Biology, School of Life Sciences, Fudan University, Shanghai, China; 2 State Key Laboratory of Plant Physiology and Biochemistry, College of Biological Sciences, China Agricultural University, Beijing, China; Stanford University School of Medicine, UNITED STATES

## Abstract

INDUCER OF CBF EXPRESSION 1 (ICE1) encodes a MYC-like basic helix-loop-helix (bHLH) transcription factor playing a critical role in plant responses to chilling and freezing stresses and leaf stomata development. However, no information connecting ICE1 and reproductive development has been reported. In this study, we show that ICE1 controls plant male fertility via impacting anther dehydration. The loss-of-function mutation in *ICE1* gene in *Arabidopsis* caused anther indehiscence and decreased pollen viability as well as germination rate. Further analysis revealed that the anthers in the mutant of *ICE1* (*ice1-2*) had the structure of stomium, though the epidermis did not shrink to dehisce. The anther indehiscence and influenced pollen viability as well as germination in *ice1-2* were due to abnormal anther dehydration, for most of anthers dehisced with drought treatment and pollen grains from those dehydrated anthers had similar viability and germination rates compared with wild type. Accordingly, the sterility of *ice1-2* could be rescued by ambient dehydration treatments. Likewise, the stomatal differentiation of *ice1-2* anther epidermis was disrupted in a different manner compared with that in leaves. ICE1 specifically bound to MYC-recognition elements in the promoter of *FAMA*, a key regulator of guard cell differentiation, to activate *FAMA* expression. Transcriptome profiling in the anther tissues further exhibited ICE1-modulated genes associated with water transport and ion exchange in the anther. Together, this work reveals the key role of ICE1 in male fertility control and establishes a regulatory network mediated by ICE1 for stomata development and water movement in the anther.

## Author summary

INDUCER OF CBF EXPRESSION 1 (ICE1) is a basic helix-loop-helix transcription factor playing multiple roles in *Arabidopsis*. It was initially identified as the activator of C-Repeat Binding Factor 3 (CBF3), a core modulator triggering cold acclimation. ICE1 also activates Flowering Locus C (FLC), a major repressor of floral transition, to delay flowering under fluctuating environmental stimuli. In normal conditions, ICE1 participates in control of stomatal development in leaves and endosperm breakdown in seeds. Here we describe a role of ICE1 in male fertility development of *Arabidopsis*. We provide evidence that ICE1 controls stomatal differentiation in the anther epidermis and thereby anther dehiscence and pollen viability as well as germination. Consequently, fertility of *ice1* mutant can be rescued by ambient dehydration. ICE1 regulates *FAMA*, one key regulator of guard cell differentiation, through direct binding to MYC-recognition elements in *FAMA* promoter. Moreover, we perform transcriptomic analysis using anther tissues and identify ICE1-regulated genes involved in water transport. These findings reveal a novel role of ICE1 in male fertility regulation through affecting water movement in the anther, which deepens our understanding of coordination between plant development and stress response, and potentially contributes to the pollination controls in crop breeding.

## Introduction

The stamen is the male reproductive organ of flowering plants and at a gross level comprises the filament and the anther [1, 2]. The late phase of stamen development including filament elongation, anther dehiscence, and pollen maturation, is an essential process in which mature pollen grains are released from locules in the dehiscent anthers, thus enabling pollination and fertilization [3]. Successful fertilization relies on the production and effective release of viable pollen [4]. Failure of anther opening (dehiscence) results in male sterility, although the pollen itself can be fully functional [5]. Anther dehiscence is a complex process involving multiple aspects, such as cellular differentiation and degradation, combined with tissue structure alteration as well as dehydration in anthers, which are also regulated by phytohormones [5–6]. A variety of mutants with disturbed anther development in the late stages have been identified in *Arabidopsis* and the corresponding genes are characterized. The genes characterized so far are categorized into two major functional groups. One is a set of regulators controlling anther structure dynamics including the anther cell layers formation (*e*.*g*., middle layer [6], tapetum [5], septum [7] and stomium [8–11]), secondary thickening in the endothecium [12–20], programmed cell death in sporophyte tissues of anthers (*e*.*g*., tapetum, septum and stomium) [4, 21], and cell wall degradation (*e*.*g*., degradation of cell wall components, such as cellulose, hemicellulose and pectin, in anther dehiscence zones catalyzed by cell wall-degrading enzymes) [22]. The other group includes genes affecting the anther physiological changes, such as water influx [23], ion homeostasis [24, 25] and carbohydrate metabolism [26–28]. Notably, most of the genes belonging to this functional group are closely related to anther dehydration. Young anthers take up water for growth during early developmental stages, while at later stages anthers and pollen undergo dehydration before dehiscence [29, 30]. The dehydration caused by evaporation through stomata and water transport in the vascular bundle promotes pollen grains maturation, anther dehiscence and filament elongation [31–33]. In addition, these two groups of genes are regulated by phytohormones. Studies on jasmonic acid (JA) biosynthetic genes [32–36], JA signaling components including *COI1* [37], *MYC* and *MYB* genes [38–43], and a JA transporter GTR1 [44] have demonstrated that JA plays essential roles in the control of timing of anther dehiscence and pollen maturation. JA positively affects stomium opening [45] and anther dehydration by regulating water transport from anther to filament [32, 46]. Auxin, generally known as a negative regulator of endothecium lignification, also functions essentially at late anther developmental stages [47–54]. Mutants with disrupted auxin biosynthetic genes or auxin responsive transcription factors are deficient in anther dehiscence, pollen maturation or filament elongation [55–58]. During the modulation of stomium opening in anther dehiscence and pollen maturation, auxin negatively controls the biosynthesis of JA [52, 56–59]. Deficiency of genes participating in any of these processes can cause anther indehiscence, which is mediated and coordinated by cell layers development and anther dehydration. In comparison, the studies with respect to genes involved in anther dehydration remain relatively limited.

INDUCER OF CBF EXPRESSION 1 (ICE1), also known as SCREAM (SCRM1), is a MYC-like basic helix-loop-helix (bHLH) transcription factor regulating plant responses to chilling and freezing stress and leaf stomata development in normal conditions. Under cold stress, ICE1 is subjected to cold-activated modification [60–63] and subsequently binds to promoters of *C-REPEAT BINDING FACTOR* (*CBF3*) [64] to enhance cold tolerance. The identified modification of ICE1 protein includes sumoylation and phosphorylation. In cold exposure, a small ubiquitin-related modifier (SUMO) E3 ligase, SAP and Miz 1 (SIZ1), facilitates SUMO conjugation to ICE1 [60] and a protein kinase, OPEN STOMATA 1 (OST1), phosphorylates ICE1 to enhance its stability and transcriptional activity [61]. Meanwhile, mitogen-activated protein kinase 3 and 6 (MPK3/6) also phosphorylates but destabilizes ICE1 in response to cold [62, 63]. ICE1 can be degraded through E3 ubiquitin ligases, high expression of osmotically responsive genes 1 (HOS1) [65] and constitutive photomorphogenic 1 (COP1) [66]. These established a well-characterized regulatory network of ICE1 in low temperature. In ambient temperature, ICE1 directly interacts with three bHLH transcription factors, SPCH, MUTE, and FAMA, to regulate stomatal differentiation in the leaf epidermis [67]. Previous studies also demonstrated that the loss-of-function mutation of *ICE1* caused early-flowering with elevated *Flower Locus C* (*FLC*) gene expression [68] and seed endosperm persistence phenotype that was also observed in the mutant of an endosperm breakdown regulator, ZHOUPI (ZOU) [69]. Thus, ICE1 functions in multiple organs at different developmental stages of plants in responses to environmental variations.

Here, we illuminate a novel role for ICE1 as a male fertility modulator in *Arabidopsis*. In the *ice1* mutant, the anther wall could not shrink to complete a sufficient anther dehiscence and anthers failed to conduct pollen release. Pollen grains from those indehiscent anthers also showed less viability and lower germination rate. Phenotypic and transcriptomic evidences indicate that the deficient anther dehiscence and pollen germination are associated with water movement and dehydration of anther wall due to the impaired stomatal differentiation as well as altered water transport and ion exchange related genes. Our work brings a new member to anther dehiscence regulators and implicates a potential link among the regulation of environmental responses, vegetative growth, floral transition and fertility development.

## Results and discussion

### Loss-of-function mutation of *ICE1* impairs fertility in *Arabidopsis*

**Fig 1:**
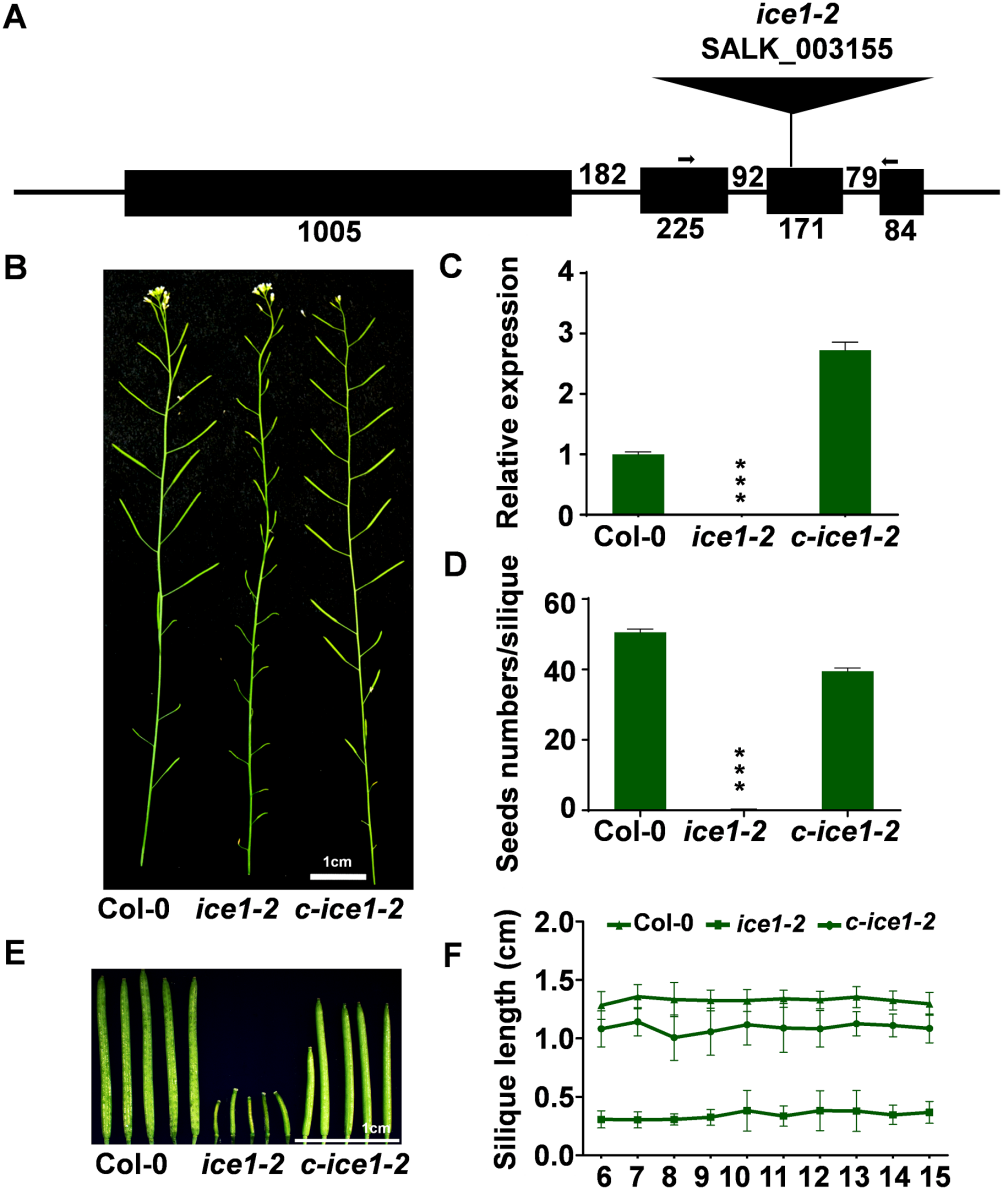
Characterization of the sterile phenotype in *ice1-2*. (A) Structures of the *ICE1* gene in *ice1-2* mutant (SALK_003155). The scaled linear map depicts four exons as boxes and three introns as bold lines between boxes. The positions of qRT-PCR primers (indicated by arrows) and T-DNA insertion are shown. (B) Morphology of reproductive growth of Col-0, *ice1-2* and *c-ice1-2* plants. (C) Relative expression of *ICE1* gene in inflorescences. The *ACTIN2* gene (AT3G18780) was an internal control. SE, n = 3, *** *p* < 0.001. (D) Comparison of seed numbers per silique of each genotype. SE, n = 32, *** *p* < 0.001. (E) Morphology of siliques from Col-0, *ice1-2* and *c-ice1-2* fresh plants. (F) Comparison of silique length of each genotype. SE, n = 14.

In the previously characterized null mutant SALK_003155 in the Columbia (Col-0) background with a T-DNA insertion in the third exon of the *ICE1* gene (Fig 1A) (named as *ice1-2*) [67], we observed reduced fertility (Fig 1B), nevertheless no information with respect to the function of *ICE1* in reproductive development has been reported. The extremely low expression level of *ICE1* was verified in inflorescences of the *ice1-2* (Fig 1C). To investigate the function of *ICE1* gene involved in plant fertility, we generated *ICE1pro*::*ICE1 ice1-2* lines, named as *c-ice1-2*. Complementation of *ICE1* expression and phenotype of reproductive development were confirmed (Fig 1B and 1C). Further characterization revealed that the *ice1-2* developed significantly shorter siliques with fewer seeds in each, while *c-ice1-2* plants showed restored phenotypes (Fig 1D–1F). In addition, *ice1-2* pistils artificially pollinated with Col-0 pollen grains were able to develop into normal siliques, while pollination using *ice1-2* pollen was failed in either Col-0 or *ice1-2* plants (S1A Fig), demonstrating that the mutant is female-fertile. Together, *ICE1* is involved in plant male fertility development and controls seed productivity. Intriguingly, another well characterized mutant *ice2-1/scrm2-1* (SAIL_808_B10) disrupting *ICE2/SCRM2*, the paralog of *ICE1* functioning similarly in cold response and leaf stomata development [70, 71], did not show any phenotype in fertility (S1B Fig), which could be due to functional redundancy or the different roles of ICE1-like transcription factors in developmental regulation.

### The *ice1* mutant is defective in anther dehiscence

**Fig 2:**
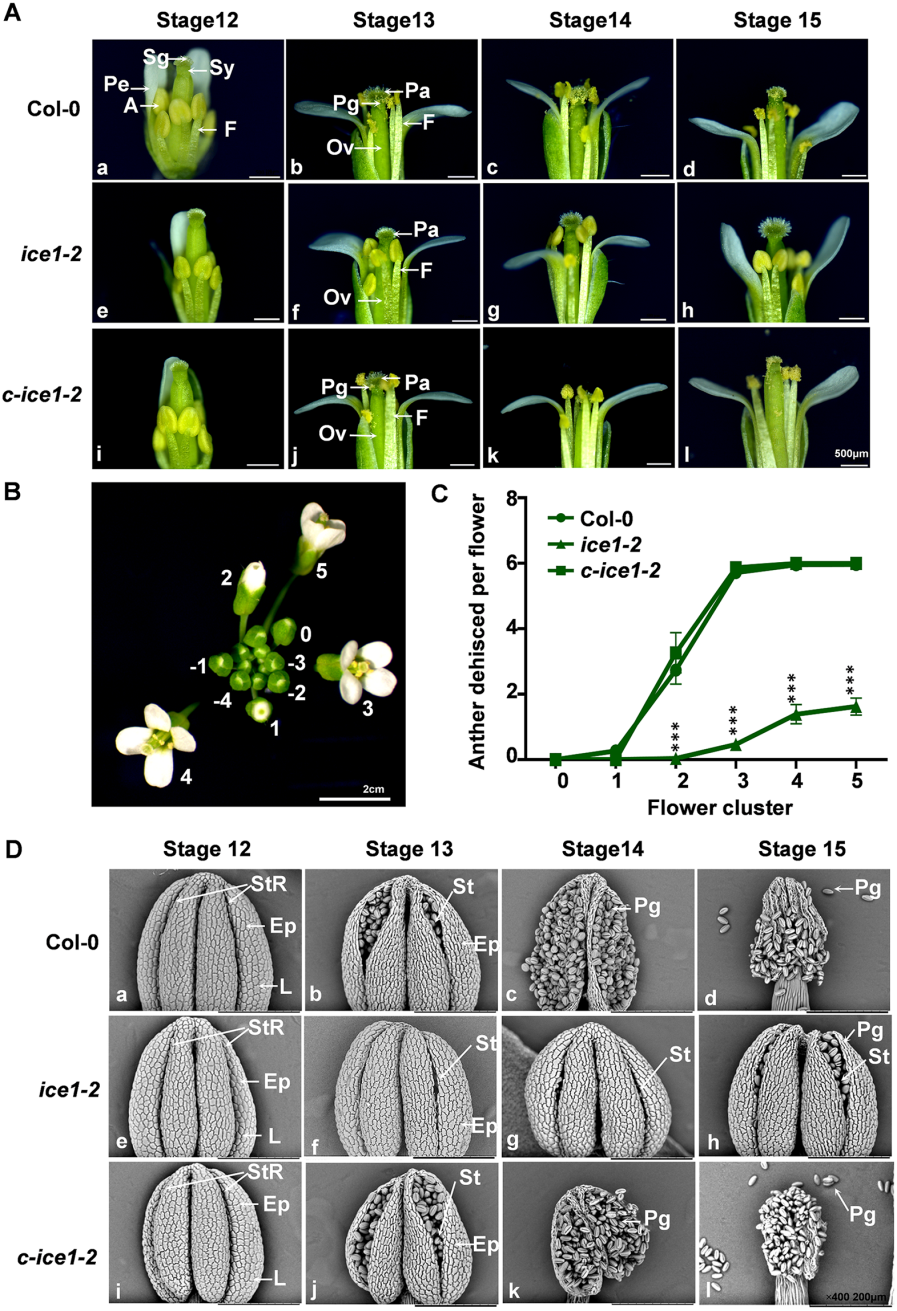
Stamen morphology and anther dehiscence in *ice1-2*. (A) Developmental series of flowers at flower developmental stage 12–15 within a single inflorescence from Col-0, *c-ice1-2* and *ice1-2*. A, anther; F, filament; Ov, ovary; Pa, stigmatic papilla; Sg, stigma; Sy, style; Pe, Petal; Pg, Pollen grain. (B) Flower cluster showing the developmental series used to quantitatively describe anther dehiscence. The number 0 indicates the beginning of flower stage 12; 2 indicates the end of stage 12; 1 (stage 12); 3 (stage 13); 4 (stage 14); 5 (stage 15); -1 (stage 11); -2 (stage 11); -3 (stage 10); -4 (stage 9). (C) The number of dehiscent anthers in plants (SE, n = 15–28 flowers, one inflorescence per plant was used, *** *p* < 0.001). (D) Scanning electron micrographs of the anther adaxial surface from flower stage 12–15. St, stomium; En, epidermis; L, locule, StR, stomium region; Pg, Pollen grain.

After a closer examination of flower anatomy using scanning electron microscopy (SEM), we observed very few pollen grains around the style or on the stigma in *ice1-2* (S2B Fig) compared with Col-0 (S2A Fig) and *c-ice1-2* (S2C Fig), thus stigmas of *ice1-2* typically were unpollinated. Besides, anthers were only occasionally open while most of them remained indehiscent in *ice1-2*. We then compared the floral development in Col-0, *ice1-2*, and *c-ice1-2* plants using light microscopy across flower development stages [45, 72]. At stage 12, no difference of anther morphology was observed in Col-0, *ice1-2* and *c-ice1-2* (Fig 2Aa, 2Ae and 2Ai). In Col-0 and *c-ice1-2*, anthers started to dehisce at stage 13, with concomitant pollen release from the locules after the full expansion of the stigmatic papilla (stage 13) (Fig 2Ab and 2Aj) and shriveling of the anther epidermis cell wall (stage 14) (Fig 2Ac and 2Ak), followed by initial stages of silique expansion and floral senescence (stage 15) (Fig 2Ad and 2Al) [1]. In contrast, most of *ice1-2* anthers did not dehisce at flower stage 13 and later stages (Fig 2Af–2Ah). Majority of the mutant anthers did not dehisce and release pollen grains until the initiation of floral senescence (stage 15) (Fig 2Ah). Based on the flower developmental series, we quantitatively analyzed the process of anther dehiscence in single inflorescences. The youngest flower with visible petals within a flower cluster was labeled as flower 1 and the next elder flower was labeled as flower 2, and so on [45] (Fig 2B). In Col-0 and *c-ice1-2* plants, more than 95% of anthers had dehisced in flower 3 (5.72 of 6 in Col-0 and 5.87 of 6 in *c-ice1-2*) and elder ones, while the dehisced anther number was significantly lower in *ice1-2* in flowers 3–5 (7.7%, 0.46 of 6 for flower 3). Even in the oldest flower 5 only 27% (1.62 of 6) of anthers were dehisced (Fig 2C). In fact, even for dehisced anthers in *ice1-2*, most of them were still not fully open like that in Col-0. Therefore, *ICE1* is required for dehiscence of anther and the decrease of fertility in *ice1-2* is related to indehiscent anthers. Further characterization of anther adaxial surface using SEM provided a closer insight into this phenotype. At stage 12 of anther development in Col-0 and *c-ice1-2* flowers, the anthers had locules filled with liquid and an indentation (stomium region) in epidermis [72] (Fig 2Da and 2Di). From stages 12 to 13, the dehiscence program was initiated from the apical toward basal parts. A stomium emerged at the apical of anther and the epidermis cells started to shrink (Fig 2Db and 2Dj). The slit on the stomium begins to widen, resulting in release of pollen at stages 14 (Fig 2Dc and 2Dk) and stages 15 (Fig 2Dd and 2Dl). In contrast, in *ice1-2* anthers the stomium slit was visible at stage 13 and stage 14 (Fig 2Df and 2Dg). However, the stomium did not rupture sufficiently even at stage 15 and epidermis cells failed to shrink to release pollen from individual anther locules to the stigma (Fig 2Dh). Hence, the *ice1* mutation disrupts the shrinkage of anther wall and prevent the release of pollen at the proper stage of pollination. Previous studies have shown that failure of anther dehiscence can be elicited by abnormal cell organization and differentiation of anther tissues [4]. The key processes affecting dehiscence include development of cell layers of the anther [6, 73], endothecium secondary thickening [12, 14], degradation of middle layer and tapetum [6, 74], septum breakdown [33, 75–77], and stomium opening [78]. To determine if there was morphological abnormality in the anther tissues, we observed transverse sections of Col-0 and *ice1-2* anthers from the emergence of dehiscence to senescence during stamen development. In both Col-0 and *ice1-2*, tapetum was visible and started to break down at anther developmental stage 10; at stage 11 endothecium started the lignification for secondary thickening, tapetum was degraded, and septum started to break down; at stage 12 the septum was degraded through a programmed cell death-like lysis to form a single locule (S3 Fig). In Col-0, stomium was open and epidermis started to shrink to release pollen grains at stage 13, and epidermis kept shrinking and releasing pollen at stage 14a. Until stage 14b all pollen grains were dispersed. In *ice1-2*, although stomium was ruptured, epidermis did not shrink and pollen grains were still covered inside the locules until stage 14b (S3 Fig). The auramine O staining in both semi-thin sections and fresh anthers at anther stage 13 also showed that no obvious difference was between Col-0 and *ice1-2* for endothecium secondary thickening that was occurred from stage 11 (S4A and S4B Fig). Whereas at stage 14 very few pollen grains were still inside anthers of Col-0 (S4Be Fig), while the *ice1-2* anthers were full of pollen (S4Bf Fig). Taken together, *ICE1* may not influence formation of anther cell layers but regulates epidermis shrinkage at the stage of pollen dispersal.

Further, the sizes of stamen and pistil tissues were also investigated using light microscopy. The filaments were fully elongated to position the anthers at the height of the stigma at flower developmental stage 14 in Col-0 and *c-ice1-2* (S5Aa and S5Ac Fig). In *ice1-2*, the stamen and style lengths were slightly shorter and the stamen/style length ratio was smaller (S5B and S5C Fig). The reduced elongation of stamen tissues is also commonly observed in mutants interrupting anther dehiscence [4]. But in *ice1-2*, the shorter stamen and pistil may not be the main reason of sterility, since the filaments were able to elongate and allowed anthers to reach stigma (S5Ab Fig).

### The *ice1* mutant shows decreased pollen viability and germination rate

**Fig 3:**
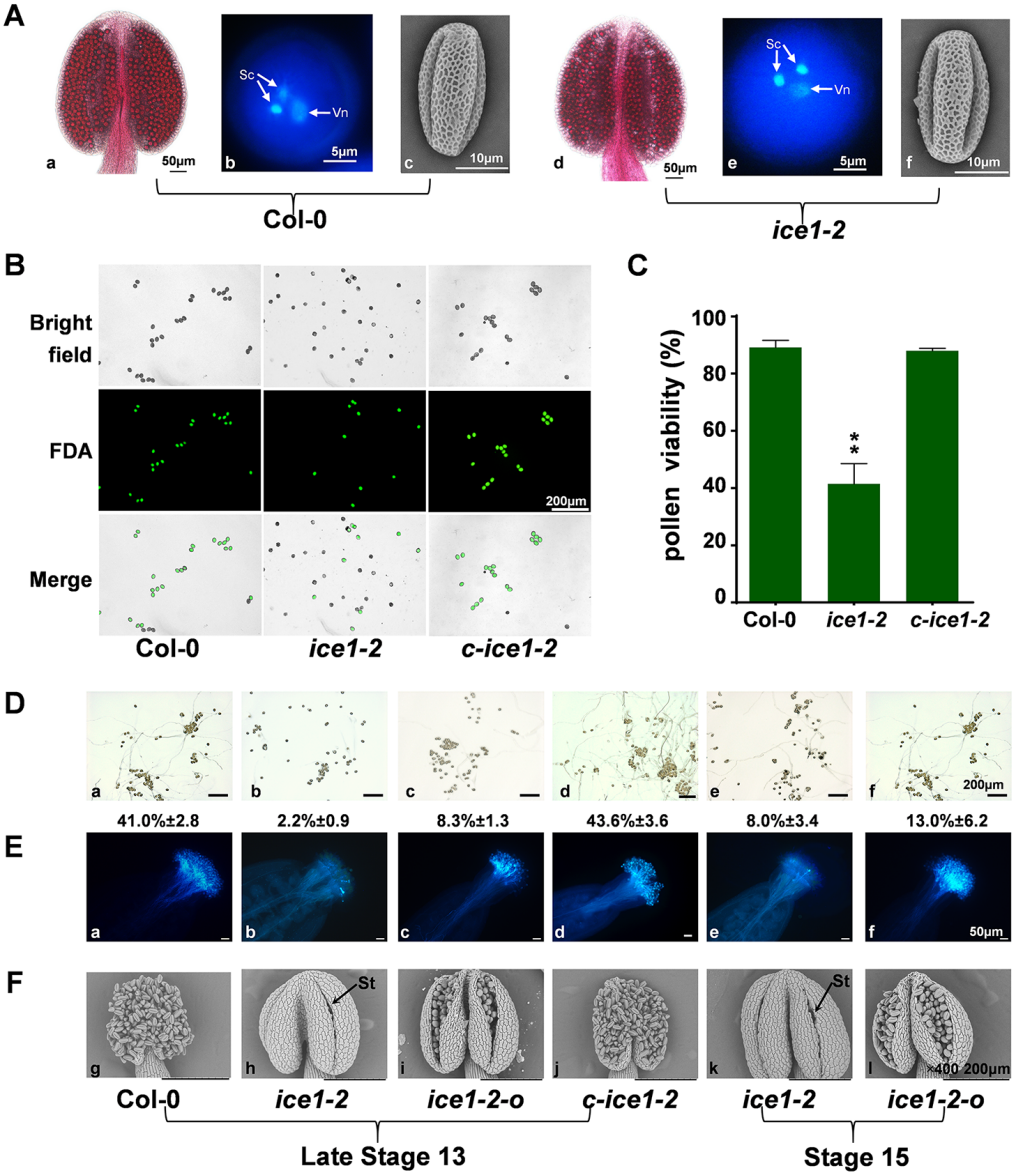
Pollen phenotypes, viability and germination analysis in *ice1-2*. (A) Alexander staining of the anther, DAPI staining of pollen at tricellular stage, scanning electron microscopy (SEM) of pollen grains from Col-0 and *ice1-2*. Vn, vegetative nuclei; Sc, sperm cells. (B) FDA (fluorescein diacetate) staining of pollen from Col-0, *ice1-2* and *c-ice1-2* at flower stage 13. (C) Comparison of viability of pollen from Col-0, *ice1-2* and *c-ice1-2*. SE, n = 5, ** *p* < 0.01. (D) The *in vitro* germination of pollen from Col-0, c*-ice1-2*, *ice1-2* and selected *ice1-2* anthers with obviously open and enlarged stomium (*ice1-2-o*) at flower stages indicated. The germination rates are listed below photographs. SE, n = 3. (E) Aniline blue-stained pistils of Col-0 flowers at 2 h after pollination with pollen from Col-0, c*-ice1-2*, *ice1-2* and *ice1-2-o* at flower stages indicated. (F) Scanning electron micrographs of the anther adaxial surface from Col-0, c*-ice1-2*, *ice1-2* and *ice1-2-o* at flower stages indicated. Arrows indicate the stomium in the *ice1-2* anther. St, stomium.

During the dehiscence of the anther, one of the key forces that open the anther comes from the swelling of pollen grains [79]. In mutants such as *apy6/7* [80], *yuc6* [81] and *ams* [82], delay or lack of anther dehiscence is due to abnormal pollen exine formation or absence of pollen. Here, the pollen development in Col-0 and *ice1-2* was examined. Similar with Col-0, *ice1-2* anthers enveloped fully differentiated pollen grains (Fig 3A). The microspores developed into tricellular pollen and the exine structure was normally formed, suggesting an intact meiotic division process and completed trinucleate stage. However, viability of *ice1-2* pollen grains was obviously lower than Col-0 and *c-ice1-2* shown by fluorescein diacetate (FDA) staining (living cell emits blue-green light [40]) at anther stage 13 (Fig 3B and 3C), indicating that the pollen maturation was influenced at the final phase. Moreover, *ice1-2* pollen grains showed a significantly lower *in vitro* germination rate compared with Col-0 at stage 13, and the germination remained poor until stage 15 (Fig 3D). Consistently, the *in vivo* germination capacity determined through pollination on Col-0 pistils also demonstrated that *ice1-2* pollen was deficient in germination (Fig 3E). Most of *ice1-2* anthers were manually opened or enlarged for collection of pollen grains. Interestingly, we noticed that when we selected the small proportion of *ice1-2* anthers with obviously open stomium and pick pollen grains exposed at the stomium area to do the pollination, the germination was rescued at both stage 13 and stage 15 (Fig 3E). Notably, even for those *ice1-2* anthers with open stomium, most of them were still half-dehiscent (Fig 3F). In the *in vitro* germination assay, hundreds of pollen grains including ones exposed at the stomium area and those enveloped inside epidermis were pooled on media. Thus, it was not surprising to see that pollen grains from *ice1-2* anthers possessing open stomium still showed low *in vitro* germination rate, which was higher than typical *ice1-2* anthers though (Fig 3D). Given the fact that pollen structure was intact and pollen grains exposed at the stomium area could germinate in pollination, the impaired pollen viability and germination in *ice1-2* might be related to abnormal anther dehiscence and dehydration.

### The impaired anther dehiscence, pollen viability and pollen germination in *ice1* mutant are due to deficiency in anther dehydration

**Fig 4:**
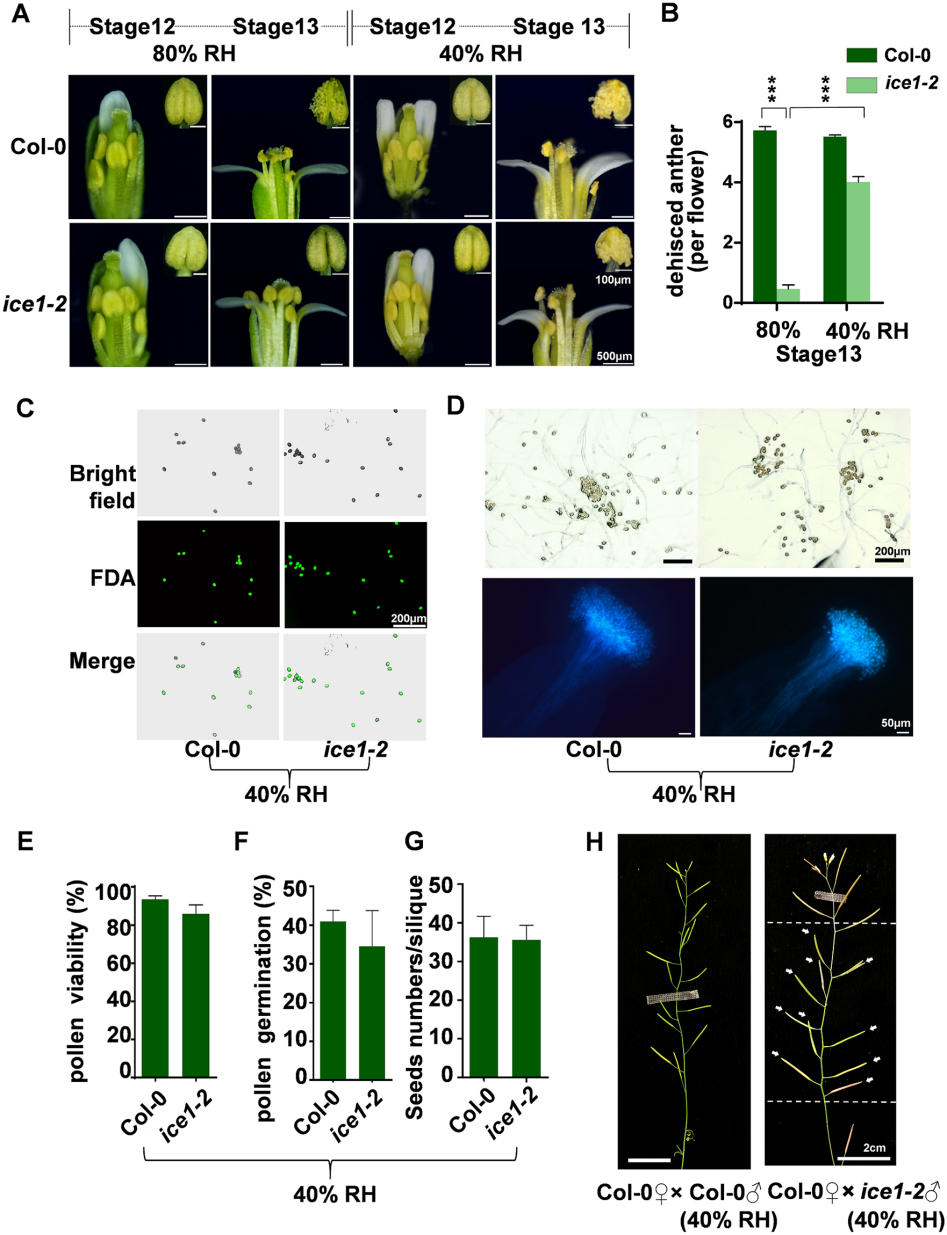
The pollen inviability, low pollen germination rate and anther indehiscence in *ice1-2* can be rescued when grown in low humidity. (A) Flowers and anthers from Col-0 and *ice1-2* plants grown under 40% or 80% relative humidity (RH), respectively. The insets (top left corner) exhibit magnification of anther phenotypes. (B) Comparison of dehisced anther numbers between Col-0 and *ice1-2* per flower at flower stage 13 under 40% and 80% RH, respectively. (SE, n = 25–292 flowers, *** *p* < 0.001). (C) FDA (fluorescein diacetate) staining of pollen from Col-0 and *ice1-2* at stage 13 under 40% RH. (D) The *in vitro* germination of pollen from Col-0 and *ice1-2* at stage 13 under 40% RH (upper row). The aniline blue-stained pistils of Col-0 flowers at 2 h after pollination with pollen from Col-0 and *ice1-2* at stage 13 under 40% RH are also shown (lower row). (E-G) Comparison of pollen viability (E) (SE, n = 5), pollen germination rates (F) (SE, n = 3), and seed numbers per silique (G) (SE, n = 20) between Col-0 and *ice1-2* grown under 40% RH. (H) Manual pollination on the Col-0 plants grown in the normal condition with pollen from Col-0 and *ice1-2* under 40% RH, respectively. Arrows indicate the normal siliques generated using *ice1-2* pollen under 40% RH.

**Fig 5:**
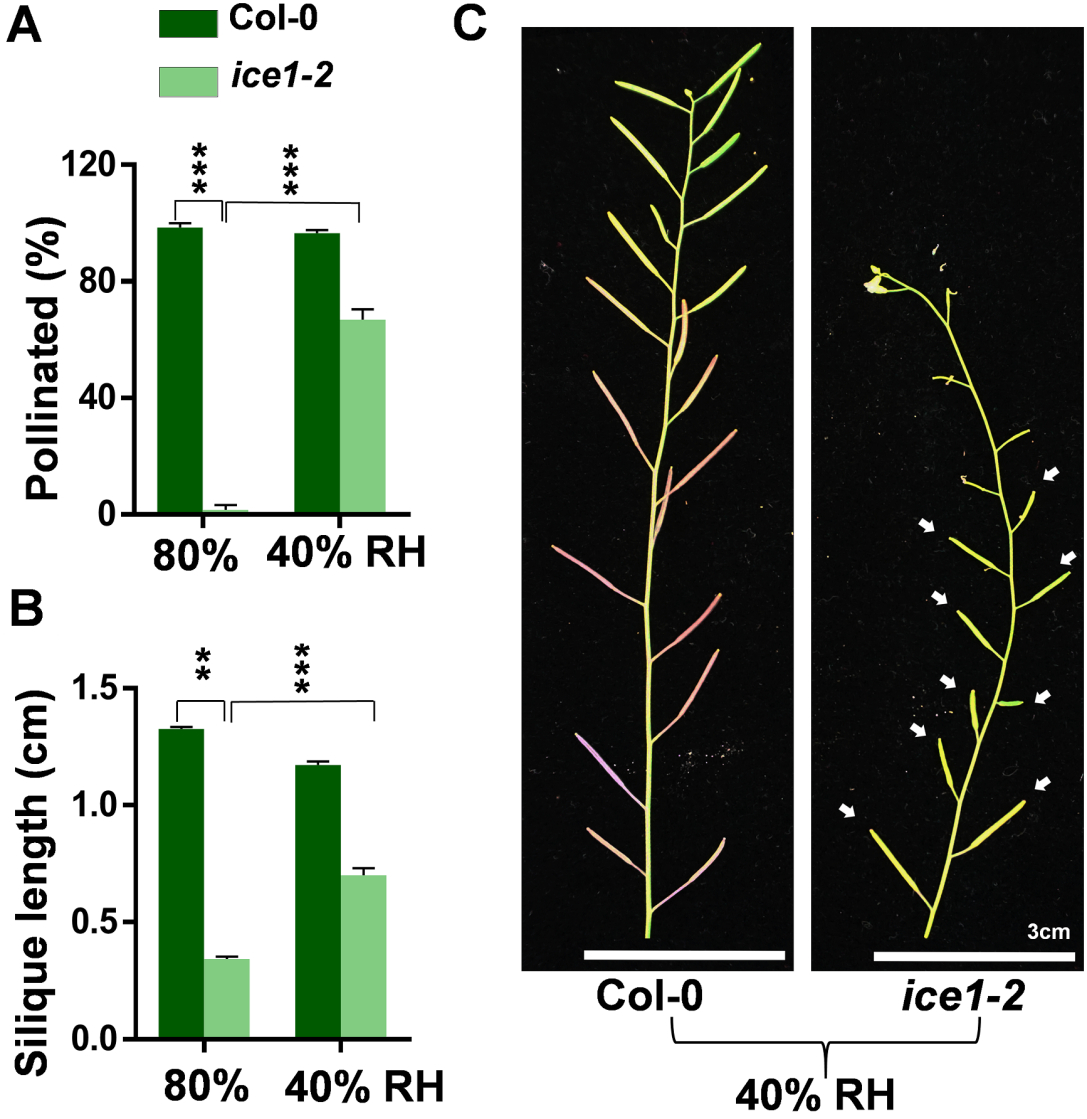
The Fertility of *ice1-2* can be rescued when grown in low humidity. (A) Comparison of pollination in Col-0 and *ice1-2* plants grown under 40% and 80% relative humidity (RH), respectively. SE, n = 62–292 flowers, *** *p* < 0.001. (B) Comparison of silique length in Col-0 and *ice1-2* plants grown under 40% (SE, n = 73) and 80% RH (SE, n = 140), respectively. ** *p* < 0.01, *** *p* < 0.001. (C) The shoots of the Col-0 and *ice1-2* plants grown under 40% RH. Arrows indicate the rescued siliques in *ice1-2*.

Water status is critical for development of pollen grains and anthers. Pollen maturation and anther dehiscence are coordinated processes involving water absorbance and dehydration of anther tissues including endothecium and epidermal cells [4, 83]. Desiccation of the anther leading to shrinkage of the outer wall provides the final force for anther opening [31]. During pollen development, pollen water content will decrease to a minimum at maturity before dispersal, and rehydrate after pollination [83]. To confirm whether the defects of anther dehiscence and pollen maturation in *ice1-2* were due to the issue of dehydration, we examined the anther dehiscence rate in different relative humidity (RH) conditions. The 80% RH environment was the normal growth condition of *Arabidopsis* plants and 40% RH was used as the dehydration treatment. The anther dehiscence rates and phenotypes were recorded at flower stage 13 that is the key stage for anther dehiscence and pollination [1]. Under 80% RH Col-0 showed higher anther dehiscence rate than *ice1-2*, while under 40% RH the *ice1-2* anther dehiscence rate was significantly increased (Fig 4A and 4B). Moreover, the deficiency of *ice1-2* in the pollen viability (Fig 4C and 4E), pollen germination (Fig 4D and 4F), and pollen function indicated by pollination on Col-0 pistils (Fig 4G and 4H) were all rescued by 40% RH treatment. Especially for pollen, *ice1-2* reached wild type levels in all three indices. As a consequence, the sterility phenotypes of *ice1-2* could be rescued by drought treatment as well (Fig 5A–5C). These further demonstrated that in *ice1-2* the anther indehiscence and impaired pollen function are due to deficiency in dehydration of anther tissues such as anther wall, which can be derived from abnormal water allocation within the stamen. These are also consistent with the previous studies showing that pollen maturation and anther dehiscence are co-regulated during water movement associated processes [83].

### *ICE1* is expressed in anther stomata and multiple flower vascular bundles

**Fig 6:**
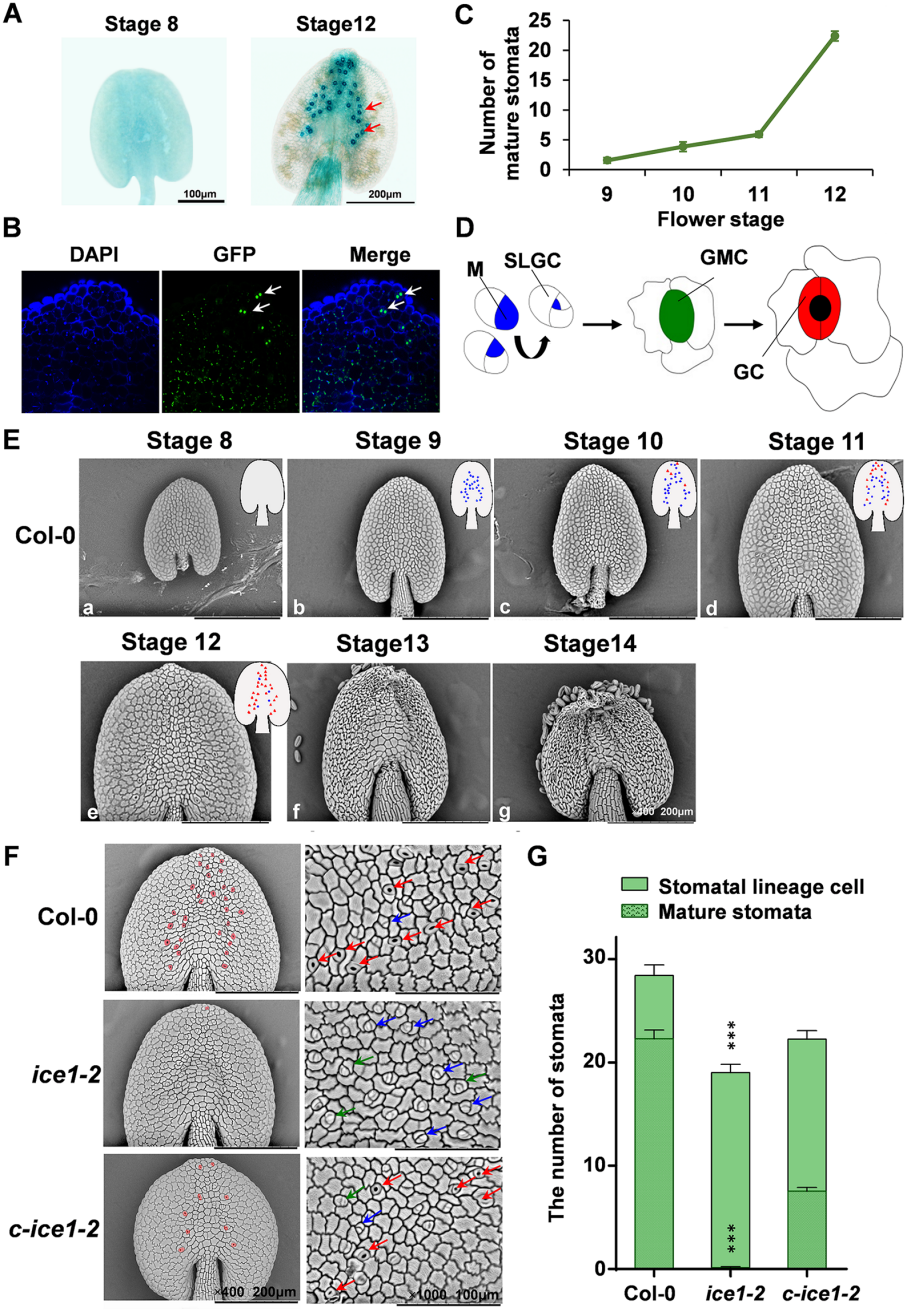
Stomata development of the anther is controlled by ICE1. (A) GUS activity driven by *ICE1* promoter is determined in the anther of flower stage 8 and 12. Strong GUS staining is shown in guard cells of stomata in the anther of flower stage 12 (indicated by red arrows). (B) Confocal images showing GFP-ICE1 accumulation (indicated by blue arrows) in the anther of flower stage 12. (C) Stomata numbers of anthers in flower stage 9–12. SE, n = 7–42 anthers. (D) Mode pattern of stomatal development in anthers. Diagram shows cell-state transitional steps within stomatal cell lineages. A subset of protodermal cells (white) assumes meristemoid mother cell (MMC) identity and executes an asymmetric entry division that creates meristemoids (M) (blue) and a sister cell, called stomatal-lineage ground cell (SLGC) (white). The meristemoids reiterate asymmetric amplifying division, but eventually differentiate into the guard mother cell (GMC) (green), which divides symmetrically once to form a stoma with differentiated guard cells (GCs) (red). (E) Scanning electron micrographs of the abaxial side of anthers at flower stage 8–14 in Col-0 (a-g). Anthers increase cell numbers from stage 8 to 9 (a-b). The stomatal lineage cells make their first appearance at about stage 9 (b). The number of stomatal lineage cells increases gradually during flower stage 9–12 (b-e). The majority of mature stomata are formed at about stage 12 (e). Inserts of anthers outline diagram show stomatal lineage cells with blue dots and mature stomata with red triangles. (F) Scanning electron micrographs of the abaxial side of anthers at flower stage 12 from Col-0, *ice1-2* and *c-ice1-2* plants. Cells colored in pink show distribution of mature stomata. Blue arrows indicate M and SLGC; green arrows indicate GMC; red arrows indicate GC. (G) Comparison of stomata numbers in Col-0, *ice1-2* and *c-ice1-2* plants at flower stage 12. SE, n = 30–42 anthers, *** *p* < 0.001).

It has been suggested that water moves out of the anther via the transport in the vascular bundle and evaporation of epidermis stomata [28, 31]. The dehydration of endothecium, connective, and locules can be partially attributable to the evaporation of water through the stomata on the abaxial surface of anthers [31]. Previous studies indicated that *ICE1* was expressed in leaf guard cells [67]. We investigated the promoter activity of *ICE1* at the stages of floral development involving anther dehiscence program events using β-glucuronidase (GUS) report system. Three independent *ICE1pro*::*GUS* transgenic lines were assayed and exhibited consistent patterns. The *ICE1* promoter showed a strong activity in the inflorescence and floral organs (S6A Fig). At approximately flower stage 10 (the petals reach the lateral stamens) [1], the style, sepals, and filaments showed strong staining, whereas no obvious GUS staining was observed in the anther tissues (S6B Fig). As the flowers developed to stage 12–15, the GUS staining remained in sepals (S6C–S6E Fig), especially vascular tissues of sepals (S6F Fig), as well as the style (S6G Fig), and turned to be much stronger in connective of anthers (S6H Fig), filaments (S6I Fig), pedicels (S6J Fig), and vascular tissues of petals (S6K Fig). In immature siliques, GUS staining was restricted to the septum, the silique tip, and the base (S6L Fig). Remarkably, although the GUS signal in the adaxial side of anthers was weak in flowers at stage 12–15, a strong staining was observed in guard cells of stomata in the abaxial side of anthers (Fig 6A), where the ICE1 protein was accordingly accumulated (Fig 6B). The water transport from anther locules to filaments and petals is essential for pollen maturation and anther dehiscence [32]. Multiple genes involved in anther dehiscence were found to be specific expressed in anther guard cells [25, 45, 84, 85], filaments [6, 32, 49], anthers and filaments junction tissues [27, 50], anther wall and vascular bundle [23]. *DAD1* strictly expressed in filaments controlling JA biosynthesis and likely water transport also regulates anther dehiscence and pollen maturation [32]. Consistent with the fact that sterile phenotype of *ice1-2* can be rescued by dehydration, the high activity of *ICE1* promoter in anther stomata and flower vascular bundles suggest a connection of ICE1 function in particular with appropriate dehydration of pollen and/or anthers.

### ICE1 regulates the stomatal differentiation in the anther

At anthesis, endothecium and epidermal cells in anther wall lose most of water via evaporation of stomata on the abaxial side of anthers [86] and osmotic retraction of water through filaments and connective tissue surrounding the vasculature [27]. Actually, in *Arabidopsis* not much information focused on stomatal development in anthers has been reported, and little attention has been paid to the role of anther stomata in anther dehiscence. Not all plant species possess stomatal pores in anther epidermis and developmental process of anther stomata depends on species [87]. In order to systematically describe the stomata development in the anther of *Arabidopsis*, we counted the number of anther stomata in flowers at stages from 9 to 12 in Col-0. The anther stomata increased from 1.57 to 5.89 at stage 9 to 11, while at stage 12 much more stomata (22.38) were identified in the anther (Fig 6C). According to the stomatal lineage model in *Arabidopsis* leaves [88], stomata differentiate via a series of cell transitions. A group of protodermal cells called meristemoid mother cells can produce meristemoids (Ms) through asymmetric divisions. Meristemoids reiterate asymmetric divisions to generate surrounding stomatal lineage ground cells (SLGCs) and eventually differentiate into guard mother cells (GMCs). One guard mother cell undergoes one time of symmetric division to produce a pair of guard cells (GCs) (Fig 6D). We used scanning electron microscopy (SEM) to perform more detailed characterization for stomata lineage in Col-0 anthers of flowers from stage 8 (before generation of stomatal lineage cells) to stage 14 (after anther dehiscence). No stomata were observed in the adaxial side of anther epidermis. In the abaxial side, cell number started to increase but no stomatal lineage cells or mature GCs appeared yet at flower stage 8 (Fig 6Ea). At stage 9, cell types were destined and stomatal lineage cells as well as few mature guard cells within top area were identified (Fig 6Eb). After that, the epidermal cells gradually expanded and more stomata turned to mature. At stage 10 and 11, mature GCs kept increasing (Fig 6Ec and 6Ed). At stage 12 with a longer duration, the number of mature GCs significantly increased, and most of stomata matured completely at the end of stage 12 (Fig 6Ee). At this moment, the anther shape was changed from oval to round and stomata gradually matured from the top to the bottom. Mature GCs were concentrated in the middle lengthways of the abaxial side in the anther epidermis (Fig 6Ee). From stage 13 to 14, the enhancing shrinkage of anther wall prompted the rupture in the adaxial side and the pollen dispersed (Fig 6Ef and 6Eg). Stomata were not present in filaments. The accumulation of matured stomata in stage 12 from the top toward the bottom in epidermis coincided the stage at which the anther wall started to shrink and then opened from the top, suggesting the role of stomata in anther dehydration and dehiscence in *Arabidopsis*.

*ICE1* has been reported as a regulator of stomatal differentiation at the surface of leaves [67], but it is unclear whether *ICE1* is involved in stomatal differentiation in anthers. Since in mature stomata of anthers *ICE1* promoter was strongly active and ICE1 protein was highly accumulated (Fig 6A and 6B), we therefore examined how *ice1-2* mutation affected stomatal development in anthers. At flower stage 12, Col-0 and *c-ice1-2* possessed abundant matured guard cells and some stomatal lineage cells, while *ice1-2* showed many meristemoids and guard mother cells but not a single mature stoma (Fig 6F and 6G). No stomata clusters or GMC-like tumors were identified either (Fig 6F). In addition, the total number of stomatal lineage cells in *ice1-2* were obviously lower than Col-0 in anthers (Fig 6G). These differed from the stomata development in *ice1-2* leaves, in which stomata clusters, GMC-like tumors aligned in parallel, and some differentiated GCs expressing mature guard cell marker E994 were present [67]. Consistently, we observed that in *ice1-2* leaves more than one third of stomata showed differentiated GCs and nearly half were immature stomata including GMC-like tumors. Stomata clusters were also recorded (S7A and S7B Fig). In comparison, *ice1-2* leaves resemble *fama* leaves in stomata development phenotype showing excessive GMC symmetric divisions and defective terminal differentiation of GCs [67], but the phenotype in *ice1-2* leaves is weaker for they can still form some differentiated GCs [67, 89] (S7A Fig). Whereas *ice1-2* anthers do not exhibit structures indicating unrestricted GMC symmetric divisions and hardly possess differentiated GCs. Thus, *ICE1* prompts stomatal differentiation in the anther in a different manner compared with that in leaves, and therefore can regulate anther dehydration to allow the dehiscence.

### The stamen-expressed and the guard cell-expressed genes were highly overlapped within ICE1-regulated gene sets

**Fig 7:**
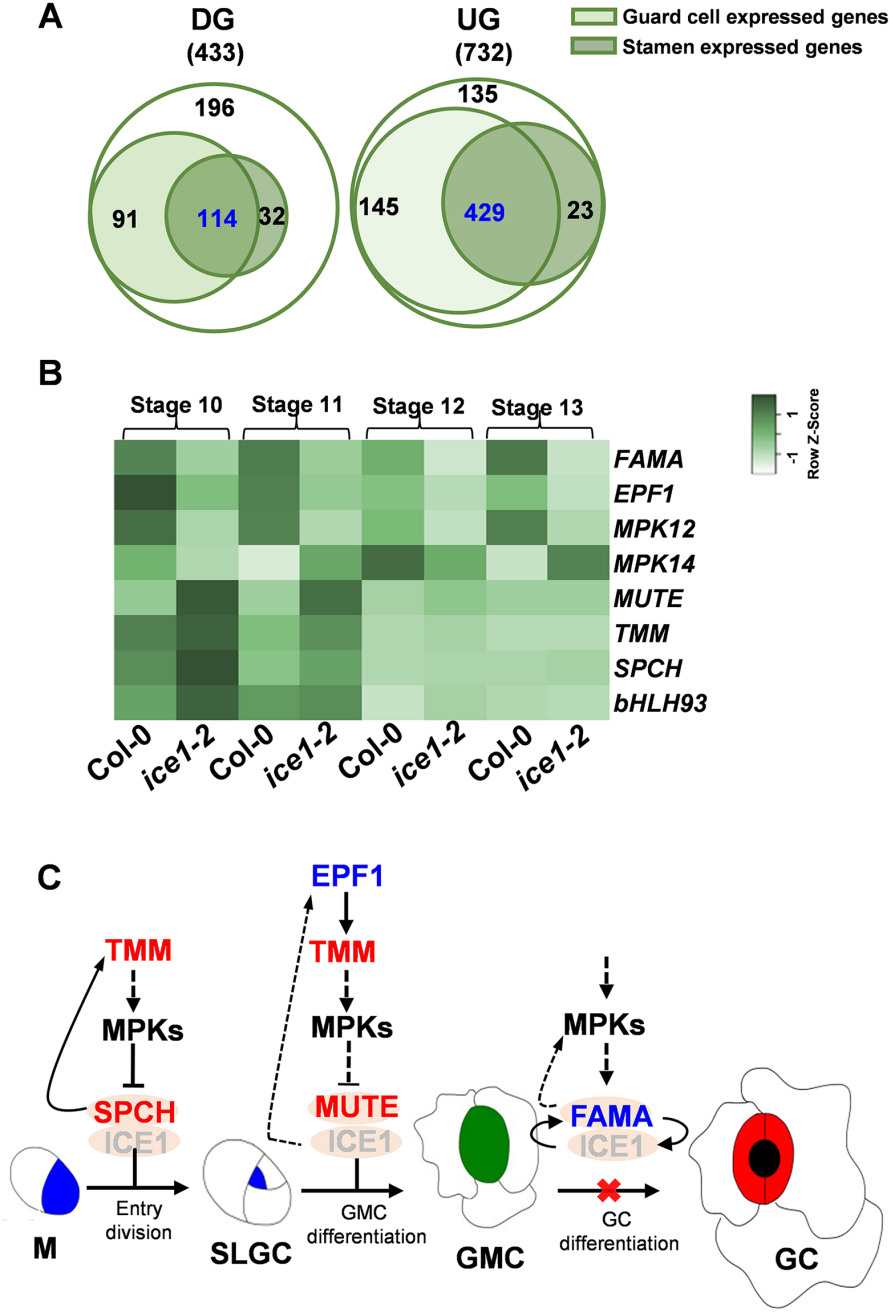
Guard cell expressed genes are overrepresented within ICE1-regulated genes in the anther. (A) Number of down- and up-regulated genes (DG and UG) in anthers at flower stage 9–13 from *ice1-2* compared with that in Col-0. The guard cell-expressed genes and the stamen-expressed genes are shown in circles of light green and dark green, respectively. (B) Heat map showing expression patterns of eight leaf stomatal development genes at the flower stage 10–13 in anthers from Col-0 and *ice1-2* measured by qRT-PCR. The gene expression profiles were normalized with *ACTIN2* gene (AT3G18780) and were plotted using Heatmapper (http://www2.heatmapper.ca/). (C) Regulatory network of stomatal development in the anther. Red shows up-regulated genes and blue shows down-regulated genes in the anther of *ice1-2*. When *ICE1* is knocked-out, the differentiation from guard mother cells (GMCs) to guard cells (GCs) is blocked.

Besides evaporation through stomata, many factors, such as signal of phytohormones, nutrient metabolism and transporters, also influence anther dehydration [23, 27, 32]. At present the direct data with respect to water content in the anther remain limited. To further investigate the effect of ICE1 underlying the phenotypes observed, we collected anthers at flower stage 9–13 covering critical time points for dehiscence and performed RNA-Seq to analyze *ICE1*-regulated genes in anthers. There were 1165 genes differentially expressed in the anther of *ice1-2* compared to Col-0, with 732 up-regulated genes (UGs) (LogFC > 1, FDR < 0.05) and 433 down-regulated genes (DGs) (LogFC < -1, FDR < 0.05) (Fig 7A and S1 Table). For corroboration of the transcriptome data, three up-regulated genes and three down-regulated genes were subjected to qRT-PCR and these expression changes showed a good agreement between RNA-seq and qRT-PCR data (S8 Fig).

Among these differentially expressed genes (DEGs), 574 UGs and 205 DGs were identified as guard cell-expressed genes according to the gene expression database (http://www.arabidopsis.org/servlets/TairObject?type=keyword&id=19990 [90] and previously published transcriptome data of the leaf stomatal lineage [91]. Meanwhile, 452 UGs and 146 DGs were detected as stamen-expressed genes through stamen gene expression database (http://www.arabidopsis.org/servlets/TairObject?type=keyword&id=20328 [92] (Fig 7A and S1 Table). There were 429 UGs and 114 DGs expressed in both the guard cell and the stamen, indicating the significantly strong overlap between genes expressed in these two tissues for ICE1-regulated DEGs (*p* < 8.405e-44 for UGs and *p* < 1.560e-20 for DGs by hypergeometric test). The overrepresentation of guard cell-expressed genes within ICE-regulated genes in the anther reflects the key role of ICE1 in the regulatory network of stomata development of the stamen, which is in line with the phenotyping results.

### ICE1 specifically binds to *FAMA* promoter to activate its transcription

**Fig 8:**
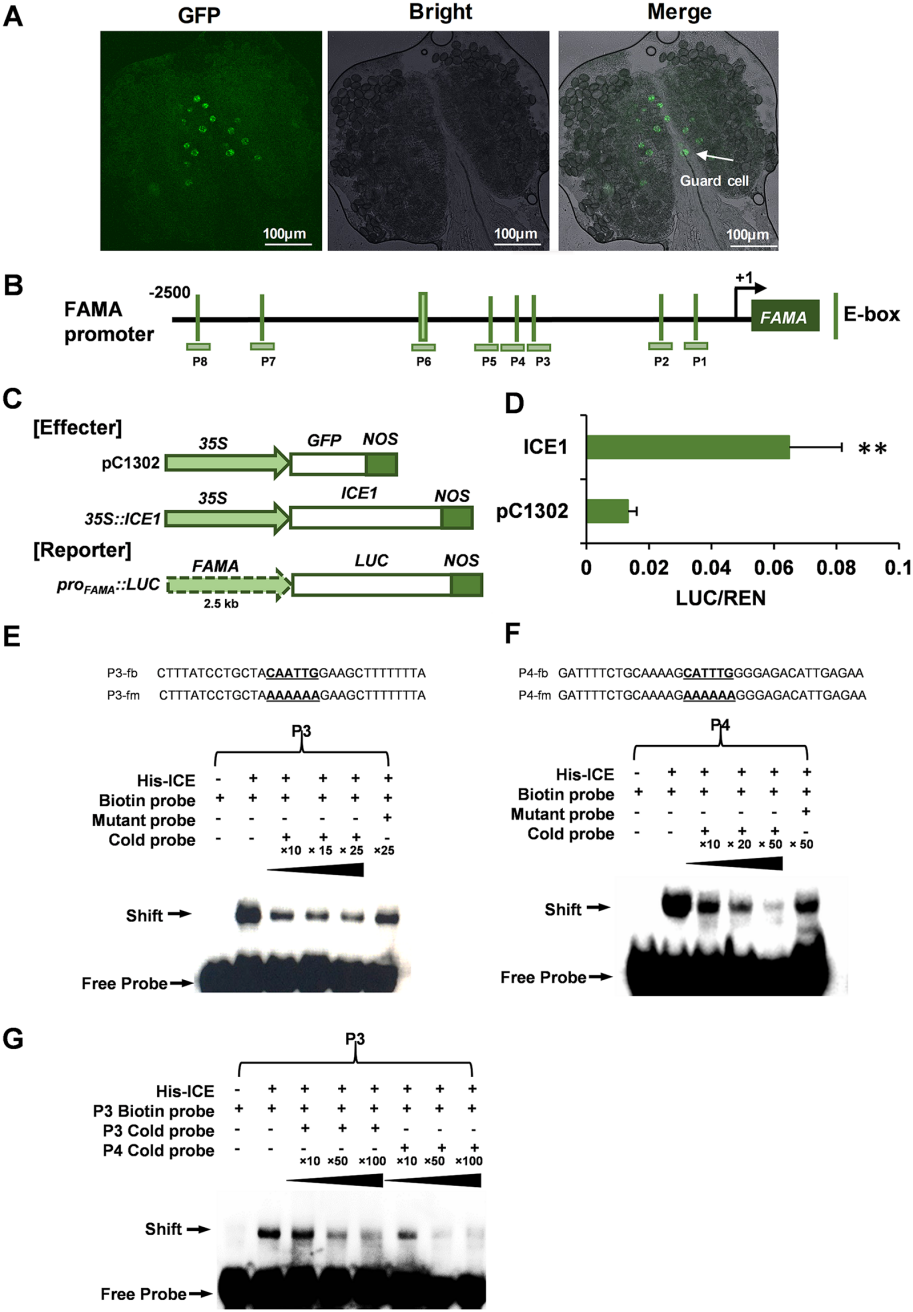
ICE1 directly binds to the promoter of *FAMA* to activate its expression. (A) Confocal images of FAMA protein accumulation (indicated by arrows) in the anther at flower stage 12 in *FAMApro*::*FAMA-GFP* plants. (B) The upstream region of 2.5 kb from transcription start site and ORF sequences of *FAMA* are shown with a black line and a blackish green box, respectively. The vertical lines indicate the E-box positions. Eight probes (P1 to P8) containing E-boxes are also exhibited. P6 contains two E-boxes. (C) Dual-LUC Assays in tobacco leaves. ICE1 driven by 35S promoter was served as the effector and LUC under control of *FAMA* promoter (2.5 kb upstream from transcription start site) was the reporter. (D) The relative activity (LUC/REN) is shown. The reporter co-transformed with pC1302 vector was used as the control. SE, n = 6, ** *p* < 0.01. (E-F) Electrophoretic mobility shift assay (EMSA) showing the binding activity of ICE1 to the probes with E-box elements at -582 to -613 bp (labeled as P3 in B) and -629 to -664 bp (labeled as P4 in B) upstream from transcription start site of *FAMA*, respectively. The sequences of P3 and P4 as well as mutated probes are listed. (G) EMSA showing the competition of ICE1-P3 interaction using P4. P4 has higher binding affinity with ICE1 than P3.

Eight of these 543 guard cell & stamen DEGs play key roles in leaf stomatal development, including four UGs (*TMM*, *SPCH*, *MUTE*, *bHLH93*) and four DGs (*FAMA*, *EPF1*, *MPK12*, and *MPK14*) [93]. The results of qRT-PCR also confirmed that the expression of these genes was differentially regulated at flower developmental stage 10–13 of *ice1-2* compared with Col-0 [83] (Fig 7B). *FAMA* and *EPF1* controlling guard cell differentiation [67, 94] were significantly down-regulated, which was in line with the impaired terminal differentiation of anther guard cells in *ice1-2*. In leaves the *ice1-2* phenotype was close to *fama*, but for anthers we could not gain *fama* materials due to its severe developmental defects [89]. The up-regulation of *TMM*, *SPCH*, *MUTE* and *bHLH93* in *ice1-2* can also be due to feedback effects (Fig 7C). Using *FAMApro*::*FAMA-GFP* plants, we observed specific accumulation of FAMA in anther guard cells (Fig 8A). Moreover, while *EPF1* promoter does not contain E-box motif (CANNTG) that is a typical binding motif of bHLH transcription factors [63], there are nine E-box elements in the *FAMA* promoter (2.5 kb from the transcription start site) (Fig 8B and S9A Fig). The *in vivo* dual-LUC assay with transient expression of ICE1 driven by 35S promoter (used as the effector) and LUC driven by truncated *FAMA* promoter fragments (used as reporters) demonstrated that in addition to protein interaction, ICE1 activated the *FAMA* transcription (Fig 8C and 8D). Further investigation using electrophoretic mobility shift assay (EMSA) showed two E-box elements located at -582 to -613 bp (labeled as P3) and -629 to -664 bp (labeled as P4) upstream from transcription start site specifically interacted with ICE1 (S9A and S9B Fig, Fig 8E and 8F). P4 exhibited an obviously higher *in vitro* binding affinity than P3 (Fig 8G). Another E-box element located at -1569 to -1600 bp (labeled as P7) also showed a weak binding with ICE1 but no competitive binding of cold probe was observed (S9B and S9C Fig), suggesting that the shift was due to a non-specific binding or the binding affinity was extremely low. P7 contains the same core sequences with P3 (S9A Fig), thus the flanking sequences may also play an important role in the ICE1 binding affinity.

The direct interaction between ICE1 and *FAMA* promoter is a novel interplay in the regulatory network of guard cell differentiation. It has been reported that *FAMA* also plays a positive role for ICE1 expression in young seedlings but does not bind to *ICE1* promoter [95]. When FAMA is associated with its promoter, it is not necessary for its own expression [89]. Given the weaker developmental defects in *ice1* than *fama*, ICE1 is unlikely necessary for *FAMA* expression. Rather, ICE1 may enhance the transcription of *FAMA* with other activators in a redundant manner, which can be a part of the regulatory network in the stomatal lineage development. However, the identification of a novel direct target of ICE1 can be potentially beneficial for breeding application.

### ICE1 regulates genes involved in water movement in the anther

**Fig 9:**
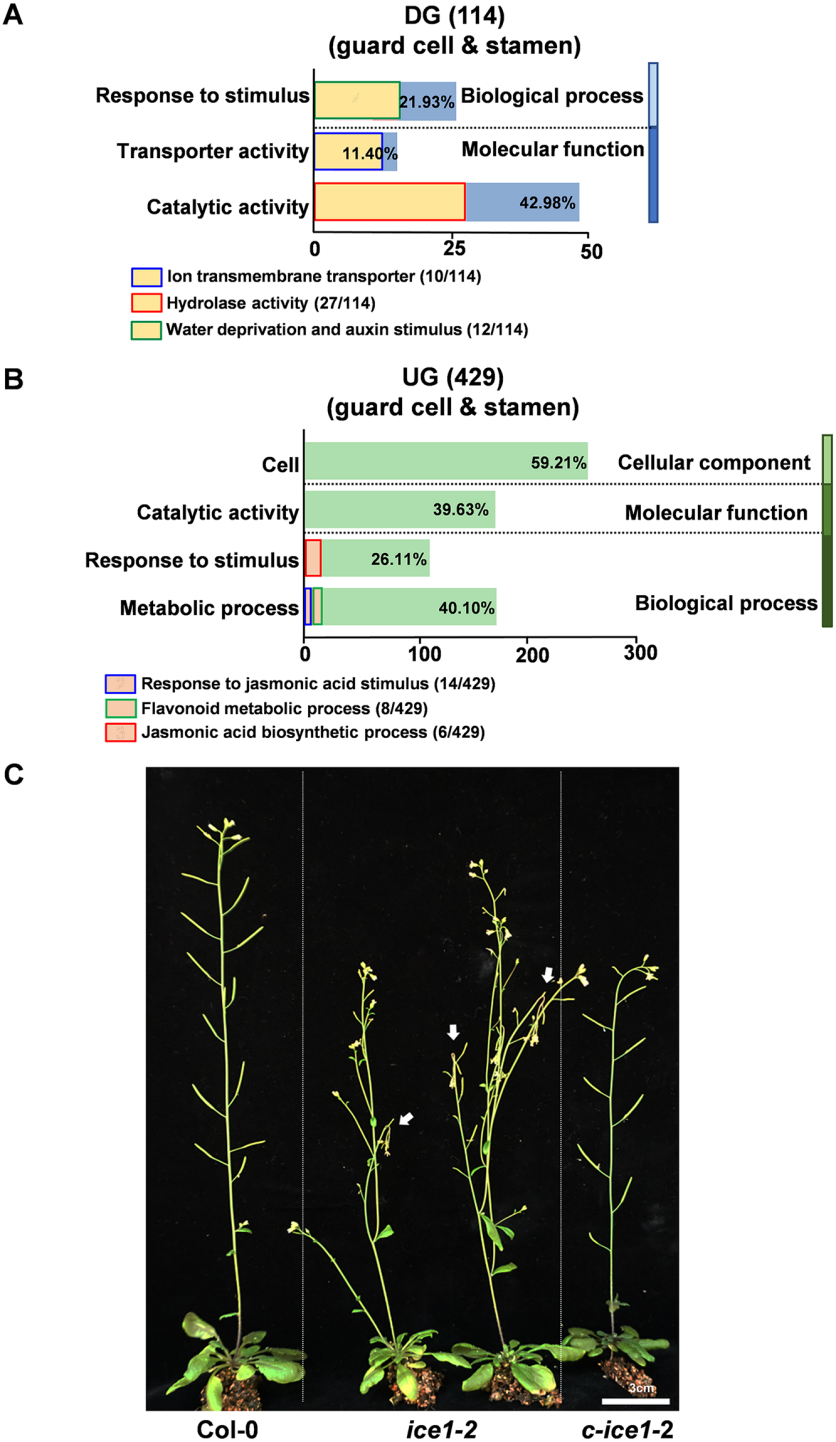
Pathway enrichment and functional category of genes regulated by ICE1 in the anther. (A-B) The enrichment analysis of down-regulated genes (A) and up-regulated genes (B) that are expressed in both of the guard cell and the stamen. The key clusters in the three categories identified by GO are shown in columns. The ratios of each cluster to the total gene number are shown with percentage. (C) Phenotype of impaired ion exchange in the *ice1-2* mutant. Arrows indicate the wilted flower buds.

Gene ontology (GO) analysis using singular enrichment provided by agriGO [96] showed that a number of ion transporters, hydrolases and dehydration associated genes were positively regulated by ICE1 in anthers (Fig 9A and S2 Table). Ion gradients or currents are critical for active water movement in the anther and they regulate the anther dehiscence and pollen germination [6, 24, 85, 97, 98]. Some mutants affecting cation homeostasis, such as *mia* deficient in a P-type ATPase cation pump [99] and *nhx1 nhx2* null in two Na^+^/H^+^ antiporters [24, 25], also failed in sufficient release of pollen from mature anthers. Twelve transporter genes, in particular genes of sugar transporters, metal transporters as well as ATPases, were down-regulated in *ice1-2* anthers (Fig 9A). Among them, *STP1* [100], *STP4* [101], *CAX3* [102] and *ACA12* [103] were expressed in leaf stomatal guard cells. The number of seeds per silique of *aca12* mutant was significantly less than that in the wild type, indicating that *ACA12* impacts plant fertility [103]. Accordingly, we observed wilted flower buds in old *ice1-2* plants, which resembled the phenotype of *nhx1 nhx2* under osmotic stress [25] (Fig 9C), suggesting that ICE1 modulates the ion exchange affecting water movement in flowers. Three glucosinolates hydrolysis related genes, *TGG1*, *TGG2*, and *TGG3*, as well as several glucosinolates biosynthesis genes, were also positively regulated by ICE1 (S2 Table). The glucosinolates are a group of secondary metabolites involved in ABA-regulated stomatal opening [104] and floral development in drought conditions [105]. The *tgg1 tgg2* mutant showed stomata with closed aperture in leaves resembling plants in the face of drought stress [106]. Thus, carbohydrate hydrolysis can also be involved in ICE1-regulated anther dehydration.

Besides, genes responding to water deprivation and auxin-mediated signaling pathways were enriched (Fig 9B, S3 Table). Two ABA-induced dehydrin genes affecting water use efficiency, *RAB18* and *LTI30* [107, 108], were remarkably repressed in *ice1-2* mutant. *RAB18* is highly expressed in guard cells, suggesting a role in stomatal function [109]. The downregulated auxin-mediated signaling genes included *SAUR41*, *GH3*.*5*, *GH3*.*6*, *BT2*, *BT5*, *IAA 32*, and *MPK12*. BT family proteins are essential during later stages of male gametophyte development [110, 111]. MPK12 is a MAP kinase that is preferentially expressed not only in leaves but also in anther guard cells [112], and positively regulates ABA [112], JA [113] and SA signaling [114] in leaf guard cells of *Arabidopsis*. It has been shown that auxin represses JA biosynthesis to control the timing of stomium opening and prevent early anther dehiscence [52]. The genes negatively regulated by ICE1 were categorized into two biological processes including JA biosynthesis and response, and flavonoids associated pathway. In the stamens and petals, JA is mainly accumulated in the filaments to regulate water transport, which sequentially triggers flower opening and anther dehiscence [32]. The JA biosynthesis or signaling deficiency can cause profoundly male sterile [4, 45]. The null mutant of *COI1*, a JA receptor, exhibited delayed anther dehiscence and produced sterile pollen [37, 45]. JA-synthesis related genes, such as *LOX2*, *AOS* and *OPR3*, affect water movement in flowers as well [45, 84] (Fig 9B and S3 Table). The interrupted transport of flavonoids leads to abnormal dehydration and dehiscence of anthers [84]. High amounts of flavonoids are also considered as endogenous auxin transport regulators that affect plant growth [115]. Here, the down-regulation of auxin signaling genes and up-regulation of JA and flavonoid related genes in *ice1-2* can be due to either active balance in regulation of water allocation or compensatory feedback consequences of failed stomium enlargement caused by abnormal water movement in the anthers and/or other floral tissues.

**Fig 10:**
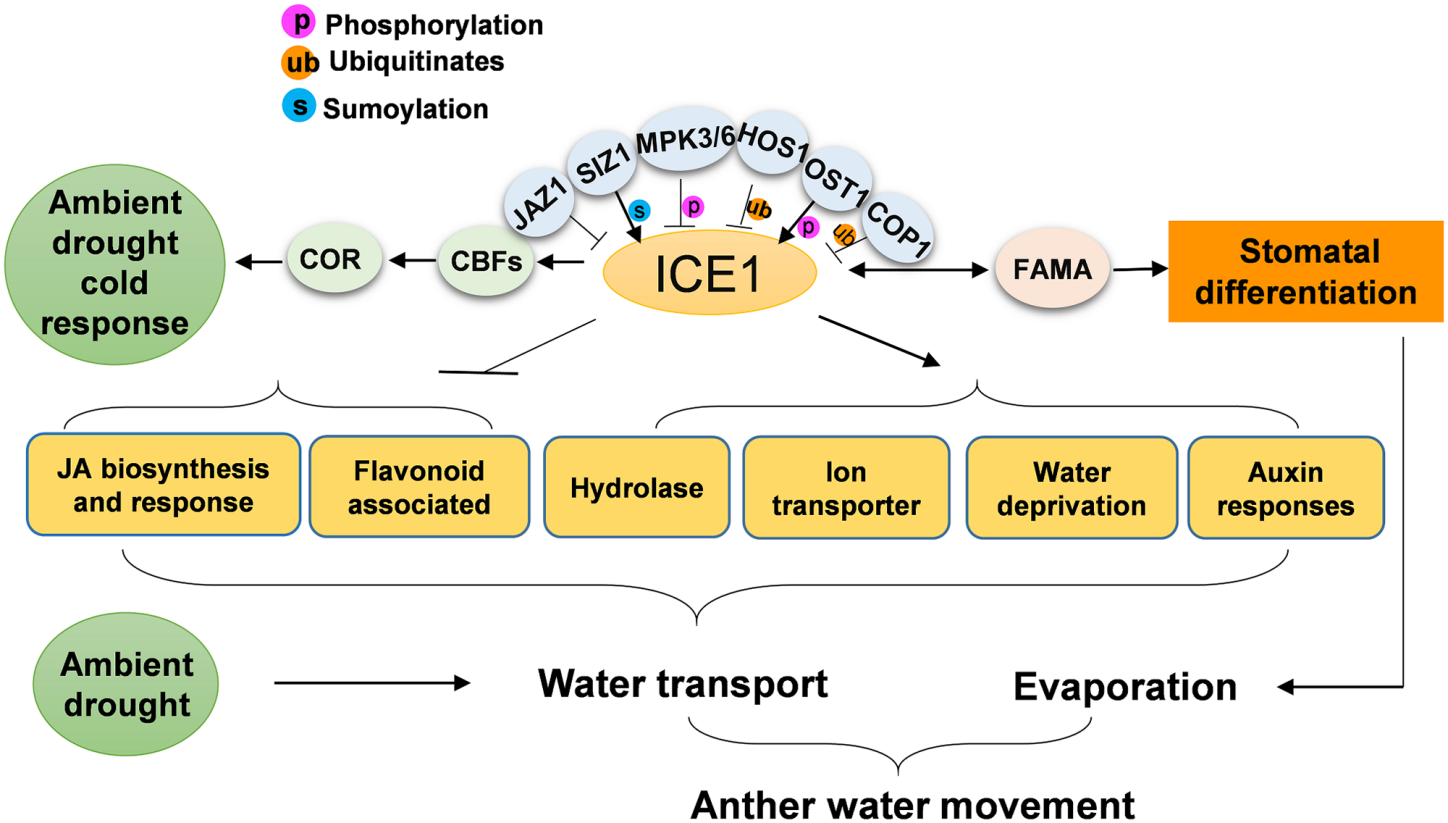
ICE1 modulates water movement in the anther. Hypothetical signal pathways deduced from GO enrichment analysis are highlighted in the yellow boxes. ICE1 modulates water transport and stomatal differentiation to control water transport in the stamen and evaporation through stomata in the anther, which can also be affected by ambient drought. On the other hand, ICE1 activates CBF signaling to protect floral tissues from drought and cold stresses.

All the identified enriched pathways in GO analysis of ICE1-regulated genes are related to water transport (Fig 10). The stomatal differentiation influencing evaporation is also controlled by ICE1. Together with the fact that dehydration rescued sterility in *ice1*, it can be demonstrated that ICE1 participates in the interaction between ambient environmental stimuli and water regulation in the anther tissues. At the same time, it has been reported that CBF3, a main target of ICE1, functions in early response to drought in flowers [105]. These can suggest a dual role of ICE1 in water-associated stress resistance and dynamic developmental processes in floral tissues. In summary, ICE1 is identified as a novel male fertility regulator in *Arabidopsis* and can be a promising target for application of molecular engineering in crop breeding.

## Materials and methods

### Plant materials

All *Arabidopsis thaliana* plants used were in the Columbia (Col-0) background. The seeds of *ice1-2* (SALK_003155) were obtained from the Arabidopsis Biological Resource Center at Ohio State University (ABRC, http://abrc.osu.edu), as previously described [67]. The *ICE1pro*::*GFP-ICE1* (*SCRMpro*::*GFP-SCRM*) transgenic line is a generous gift from Pro. Keiko Torii (Department of Biology, University of Washington). The *FAMApro*::*FAMA-GFP* transgenic line is a generous gift from Ph. D. Xiaolan Chen (School of Life Sciences, Yunnan University).

To generate *ICE1pro*::*GUS* lines, a 2578bp upstream region from the start codon was amplified by PCR from *Arabidopsis* Col-0 genomic DNA and cloned into T-vector pMD-19T (TaKaRa). After the DNA sequences were confirmed, the promoter region was cloned into pCAMBIA1301 (CAMBIA, Australia) using the method as previously described [63]. Primers were AtPICEF-PstI (5’-TActgcagGGACCACCGTCAATAACATCG-3’); AtPICER-NcoI (5’-TTccatggGCCAAAGTTGACACCTTTACC-3’). The *ICE1pro*::*GUS* plasmid was electroporated into *Agrobacterium tumefaciens* strain GV3101 (WEIDI), which was used to transform the Col-0 plants by the floral dipping method [116].

For complementation of *ice1-2* mutant, the *ICE1* upstream region and open reading frame were amplified and subcloned into pCAMBIA1302 vector using primers AtPICEF-PstI; AtPICER-NcoI; AtICE1F-SpeI (5’-ATactagtGATCATACCAGCATACCCTGC-3’); AtICE1-BstEII (5’-TTggtaaccTCAGATCATACCAGCATACCC-3’). The *ICE1pro*:: *ICE1* fusion construct was then introduced into *ice1-2/+* plants by the floral dipping method [116].

### Plant growth and drought treatment

Plants were grown in greenhouses under long day conditions (16 h light/8 h dark) at 22°C. The dehydration experiments were performed as previously described with some changes [105]. In brief, two treatments were carried out. One was the standard condition with 80% soil moisture and 80% air relative humidity. The other was drought condition with 40% soil moisture and 40% air relative humidity. Pots were arranged according to a randomized design and their positions were changed daily. Seeds were stratified in a cold room for 2 d at 4°C in the dark. Plants were grown in standard condition until the moment just after bolting (the main shoot was about 1 cm high). When the drought treatment was started, plants were transferred into the growth chamber (RXZ-436B-LED, Ningbo Jiangnan instrument factory, China). The soil moisture was maintained by daily weigh and watering until harvest.

### Pollen germination tests

Pollen germination analysis was conducted mainly as previously described [32]. The *in vitro* assay was performed on pollen germination media using pollen isolated from flowers at designed stages. For pistil pollination, pollen grains from flowers at designed stages were hand-pollinated on Col-0 pistils. The pollinated pistils were subjected to aniline blue staining or kept growth for characterization of siliques and seeds. For *ice1-2* mutant the stomium was manually enlarged for releasing pollen or picking the pollen grains using dissecting needles.

### Semi-thin sectioning and staining

Inflorescences of Col-0 and *ice1-2* mutant plants were collected, fixed and dehydrated as previously described [117]. The Technovit resin-embedded blocks were sectioned to a thickness of 1.0 μm slice using a motorized RM2265 rotary microtome (Leica) with a glass knife, and then heat-fixed on glass slides. After staining with 0.05% Toluidine Blue for 15–30 min, the sections were photographed under the Microscope Axio Scope.A1 (Carl Zeiss MicroImaging) with bright field after rinsing and drying. Lignin in tissue was visualized with 0.01% fluorescent brightener (Sigma) for 30s, then mounted with 0.001% auramine O (BBI Life Sciences) and observed by Microscope Axio Scope.A1 (Carl Zeiss MicroImaging) under GFP channel.

### Light and fluorescence microscopy

Fluorescence microscopy was performed using a Leica confocal laser-scanning microscope (Leica TCS SP8, Leica Microsystems, Wetzlar, Germany) equipped with a 10× Leica HC PL APO objective. The lignified cells and GFP fusion protein were observed with 488 nm excitation/ 510-540nm emission.

Inflorescences and anthers were collected and photographed under a SteREO Discovery V8 dissecting microscope (Carl Zeiss MicroImaging) using a SPOT FLEX digital camera (Diagnostic Instruments). Pollen from anthers stage 13–14 [72] were collected and incubated in Fluorescein Diacetate (FDA) (Solarbio) solution (FDA (5mg/ml) in acetone and diluted by 20% sucrose to 0.1mg/ml) for 5min [118], and photographed under Microscope Axio Scope.A1 (Carl Zeiss MicroImaging) under DAPI channel with an Axio Cam HRc camera (Carl Zeiss MicroImaging).

### Scanning electron microscopy (SEM)

For SEM analysis, tissues were dissected under anatomical lens (SMZ-161-BLED, Motic, China) if needed, then immediately mounted on aluminum stubs for SEM. For leaf tissues, small pieces (d = 8 mm) of leaves from about 5-week-old plants were cut, fixed, dehydrated and coated as previously described [106]. These images were taken with scanning electron microscope TM3000 (TM3000 Tabletop Microscope, HITACHI, Japan).

### GUS assay

For histochemical GUS activity analysis, tissues were immersed in GUS staining buffers with vacuum infiltration and destained with 75% ethanol as previously described [119]. The GUS activity was observed with Microscope Axio Scope.A1 (Carl Zeiss MicroImaging).

### Transient transcription dual-luciferase assays

Coding regions of ICE1 were cloned into the pCAMBIA1302. The promoter sequences of FAMA were PCR amplified and inserted into the pGreenII 0800-LUC vector, using primer pFAMAF-PstI 5‘-TGCACTGCAGTTTGGAAATTGATTTTGGGA-3’ and pFAMAR-SacII 5’-TCCCCGCGGGAGTAAGCATCACCAA-3’. After sequencing, all the constructs were transformed into GV3101 Agrobacteria, while the pGreenII-0800 constructs were co-transformed with pSoup-P19. The mixture of cells containing constructs with protein and promoter was infiltrated according to the published method [120]. The luciferase activity of *Nicotiana benthamiana* extracts was determined using the dual-luciferase assay kit (Promega) and then detected by a Synergy 2 multimode microplate (BioTek) as described previously [120]. All tests were performed with three biological replicates and five technical replicates per assay.

### Electrophoretic mobility shift assay

The electrophoretic mobility shift assay (EMSA) was performed as previously described [61]. In brief, the His-ICE1 recombination protein was expressed in E. coli induced by 1 mM IPTG at 37°C for 3 h and purified through sonication and His sepharose beads (Amersham Biosciences). EMSA was conducted using the Lightshift Chemiluminescent EMSA Kit (Pierce) with biotin-labeled and cold probes. Probe sequences were listed in S9A Fig.

### Quantitative RT-PCR

Total RNA was extracted by RNApure Plant Kit (CWBIO) according to the manufacturer’s protocol. cDNA was reverse-transcribed using PrimeScript RT reagent Kit with gDNA Eraser (Perfect Real Time) (TaKaRa). SYBR Premix Ex Taq II (TaKaRa) was used for qPCR on a ABI StepOne Plus real-time system (Life Technologies). qRT-PCR was performed in triplicate and data were collected and analyzed with ABI STEPONETM software version 2.1 [121]. Various gene specific signal was normalized relative to *ACTIN2* gene (At3G18780) expression. The primer sequences were listed as follows:

ACTIN2-Forward, 5’-CTTGCACCAAGCAGCATGAA-3’
ACTIN2-Reverse, 5’-CCGATCCAGACACTGTACTTCCTT-3’
ICE1_q3-Forward, 5’-CAACTTCATCAAGCTTCCATCCGTT-3’
ICE1_q3 Reverse, 5’-GCTGTATCGAAAAGCACTGCTTTGA-3’
TGG1-1-Forward, 5’-TCCTCAGTAAAGTCATCAAGGAGA-3’
TGG1-1-Reverse, 5’-AGACGCTTGAGCGGAGTAGA-3’
TGG2-1-Forward, 5’-TCCGCAAGGCCATCAAGGA-3’
TGG2-1-Reverse, 5’-AACGACTGGTACCATAAGCCA-3’
CYP83B1-Forward, 5’-GAGACGCAAGCACTTTTGGG-3’
CYP83B1-Reverse, 5’-TAGGGCGGTTAGGGTCAAGA-3’
GSTF9-1-Forward, 5’-GTTCCTGCTGTTGTTGACGG-3’
GSTF9-1-Reverse, 5’-AGTGGTCGCTTCCACATCAA-3’
SOT17-1-Forward, 5’-TTCTTCGTCTGCAGCTACCC-3’
SOT17-1-Reverse, 5’-AACGCTTGGGAAAAACGGGA-3’
ABI2_1-Forward, 5’-TGCAACGGTGAATCTAGGGT-3’
ABI2_1-Reverse, 5’-CCGTTGATTTCATCTCCGGC-3’
MUTE-1-Forward, 5’-CCAGACAATCGAGCCATCCA-3’
MUTE-1-Reverse, 5’-CCCACGATTCGCCTAGAGAC-3’
TMM-1-Forward, 5’-CAGTCTTCGGGTCCTTCACC-3’
TMM-1-Reverse, 5’-TCTCGAACGGTACTGGTCCT-3’
SPCH-1-Forward, 5’-CTCCGACAGCTGCATCTACA-3’
SPCH-1-Reverse, 5’-TTCTCCGGTTACGTTCCACG-3’
FAMA-1-Forward, 5’-TTTCAAGAAGAAGGCCGGGAC-3’
FAMA-1-Reverse, 5’-CCAGGTTAGAGCTTCCAGATATGTT-3’
EPF1-1-Forward, 5’-CCAACATCCTCCCATCCAAGT-3’
EPF1-1-Reverse, 5’-CGTGTGAGCAATCTGGCAAC-3’
MPK12-1-Forward, 5’-TCTGTTGGCTGCATACTCGG-3’
MPK12-1-Reverse, 5’-CGATAGCCGTAGTGGGCATT-3’
MPK14-1-Forward, 5’-GGCATGTGAGACACGAAAACG-3’
MPK14-1-Reverse, 5’-TCGCGATGAAGGATGTTTGC-3’
bHLH93-1-Forward, 5’-TCCGATCCATCGTCCCAAAA-3’
bHLH93-1- Reverse, 5’-TCCTCGTCTCTACGATCTATTTCA-3’

### RNA sequencing and data analysis

Anthers at flower stages 9–13 from Col-0 and *ice1-2* plants were collected and immediately frozen in liquid nitrogen. Total RNA was extracted using RNAeasy Plant Mini Kit (Qiagen, Valencia, CA) according to the manufacturer’s protocol. Around 2 μg of total RNA with an A260/280 value of 1.8–2.0 was used to prepare the libraries, which were subjected to paired-end (2 x 100 bp) sequencing in the Illumina Hi-seq 2000 system (Illumina Inc.). The RNA-seq analysis was performed as previously described with modifications [121]. In brief, raw reads were cleaned up with Trim Galore (https://www.bioinformatics.babraham.ac.uk/projects/trim_galore/) and mapped to the *Arabidopsis* genome (TAIR10) by TopHat2 [122], then further assembled using StringTie and Cufflinks-CuffMerge [123]. The read counts for each gene was calculated by HTSEQ v.0.6.0 [124] and the expression level was normalized as Fragments Per Kilobase of transcript per Million mapped reads (FPKM). The differential expression analysis was performed using DEGseq2 [125]. Differentially expressed genes (DEGs) were selected when Log2 Fold-Change (Log2FC) > 1 or < -1, and False Discovery Rate (FDR, Benjamini-Hochberg adjusted P-value) < 0.05. The RNA-Seq data have been uploaded to the National Center for Biotechnology Information Sequence Read Archive under accession numbers GSE107260.

### GO analysis

Gene ontology annotation and enrichment analysis was performed on agriGO, a publicly accessible analysis tool and database (http://bioinfo.cau.edu.cn/agriGO). Genes that express in guard cell or stamen were obtained by matching the annotated accessions to the annotation list under key word ID PO: 000293 (express in guard cell, http://www.arabidopsis.org/servlets/Search?type=annotation&action=search&kw_id=19990&kw=guard%20cell&scope=term) and PO:0006472; PO:0006441 (express in stamen, http://www.arabidopsis.org/servlets/Search?type=annotation&action=search&kw_id=20328&kw=stamen&scope=term).

## Supporting information

S1 Fig The female fertility of *ice1-2* and phenotype of *ice 2–1*. (A) Manual pollination on Col-0 or *ice1-2* pistils using Col-0 or *ice1-2* pollen. Arrows indicate the normal siliques generated by pollination on *ice1-2* pistils with Col-0 pollen. (B) Structures of the *ICE2* gene in the *ice2-1* mutant (SAIL_808_B10). Normal fertility was observed in *ice2-1* plants under normal growth conditions.
(TIF)

S2 Fig Characterization of the stamen in *ice1-2*. Scanning Electron Microscope (SEM) of flowers from Col-0 (A), *ice1-2* (B) and *c-ice1-2* (C) at flower stage 14. The pollen grains were released from the dehisced anther locules in Col-0 and *c-ice1-2*. The *ice1-2* pollen grains failed to be released to receptive papillae on the stigma. A, Anther; F, filament; Ov, ovary; Pg, pollen grain; S, sepal; Sg, stigma; Sy, style.
(TIF)

S3 Fig Anther developmental process in *ice1-2*. Semi-thin cross sections of anthers from Col-0 and *ice1-2* at anther stage 10-14b were stained with toluidine blue. Ep, Epidermis; En, Endothecium; T, Tapetum; StR, stomium region; St, stomium; Sm, septum; Fb, fibrous bands; C, Connective; V, Vascular bundle; Pg, pollen grains.
(TIF)

S4 Fig The endothecium lignification of anthers in *ice1-2*. (A) Transverse sectioning of anthers at anther stage 10–13 with auramine O staining. Arrows indicate the positions of endothecium lignification. (B) Fresh anthers at stage 14 with auramine O staining. Secondary thickening is visible in the endothecium (arrows indicated). (a) The anther from Col-0; (b) the anther from *ice1-2*; (c) Close-up of (a); (d) Close-up of (b); (e) Photographed by bright-field microscopy of (a); (f) Photographed by bright-field microscopy of (b). Ep, Epidermis; En, endothecium; Pg, pollen grains.
(TIF)

S5 Fig Characterization of length of the stamen and the style in *ice1-2*. (A) Phenotypes of the stamen and style in Col-0 (a), *ice1-2* (b) and *c-ice1-2* (c) at flower developmental stage 14. (B) Stamen and style lengths were measured from microscopy pictures (SE, n = 30–39 styles and 119–146 stamens, *** *p* < 0.001). (C) Ratio of filament/pistil according to length data shown in (B) (SE, n = 119–146, *** *p* < 0.001).
(TIF)

S6 Fig ICE1 promoter-driven GUS expression pattern in flower tissues. (A) Inflorescence. (B) Flower at flower stage 10. (C) Flower at stage 12. (D) Flower at stage 14. (E) Flower at stage 15. (F) Sepal at stage 14. (G) Pistil at stage 14. (H) Adaxial side of the anther at flower stage 12. (I) Filament at stage 14. (J) Pedicel at stage 14. (K) Petal at stage 14. (L) Silique.
(TIF)

S7 Fig Stomatal development of *ice1-2* in leaves. (A) Scanning electron micrographs of stomata from abaxial leaf surface. (a) Mature stomata in Col-0. Yellow brackets show stomatal cluster (b), paired differentiated guard cells (c), and immature stomata (d) in *ice1-2*. The differentiated guard cells in Col-0 (e) and *ice1-2* (f) are also shown. (B) Comparison of proportions of different stomatal types in leaves between Col-0 and *ice1-2*.
(TIF)

S8 Fig The qRT-PCR verification of RNA-seq data. Six genes were selected for comparison of RNA-seq and qRT-PCR results. For RNA-seq data, ** FDR < 0.01, *** FDR < 0.001. For data of qRT-PCR, SE, n = 3, * *p* < 0.05, ** *p* < 0.01, *** *p* < 0.001. Three independent experiments were carried out with similar results.
(TIF)

S9 Fig Electrophoretic mobility shift assay (EMSA) showing interaction of ICE1 with nine E-box elements in 2.5 kb upstream from transcription start site of *FAMA*. (A) Probe sequences containing nine E-box elements are listed. P6 contains two E-boxes. (B) Binding results of ICE1 to eight probes. P3, P4 and P7 showed binding activity. P3 and P4 exhibited competition by cold probes while P7 did not show competition. (C) P7 did not show competition by cold probes with high concentration.
(TIF)

S1 Table Full list of genes that were differentially expressed with statistical significance (FDR < 0.05) by at least 2-fold in comparison of *ice1-2* vs Col-0 in the anther at flower stage 9–13. Genes expressed in the guard cell and the stamen are labeled.
(XLSX)

S2 Table Down-regulated genes that are enriched in GO annotations. (DOCX)

S3 Table Up-regulated genes that are enriched in GO annotations. (DOCX)
