# Supplementary figures and images for: INDUCER OF CBF EXPRESSION 1 is a male fertility regulator impacting anther dehydration in Arabidopsis
Source: PLoS Genet. 2018 Oct 4;14(10):e1007695. doi: 10.1371/journal.pgen.1007695 (PMC6191155; doi:10.1371/journal.pgen.1007695)

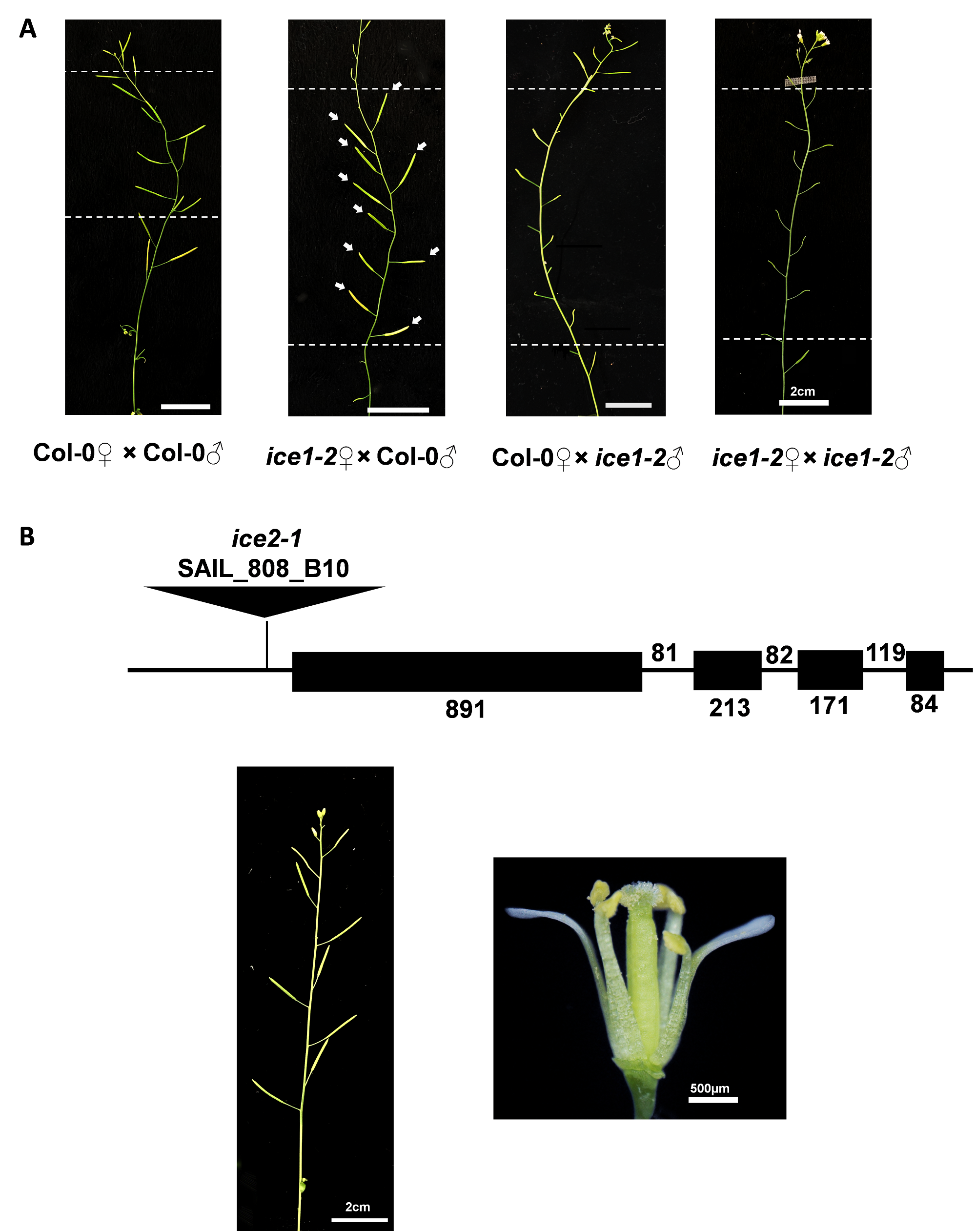

Supplement: S1 Fig — (A) Manual pollination on Col-0 or ice1-2 pistils using Col-0 or ice1-2 pollen. Arrows indicate the normal siliques generated by pollination on ice1-2 pistils with Col-0 pollen. (B) Structures of the ICE2 gene in the ice2-1 mutant (SAIL_808_B10). Normal fertility was observed in ice2-1 plants under normal growth conditions. (TIF) [file pgen.1007695.s001.tif]

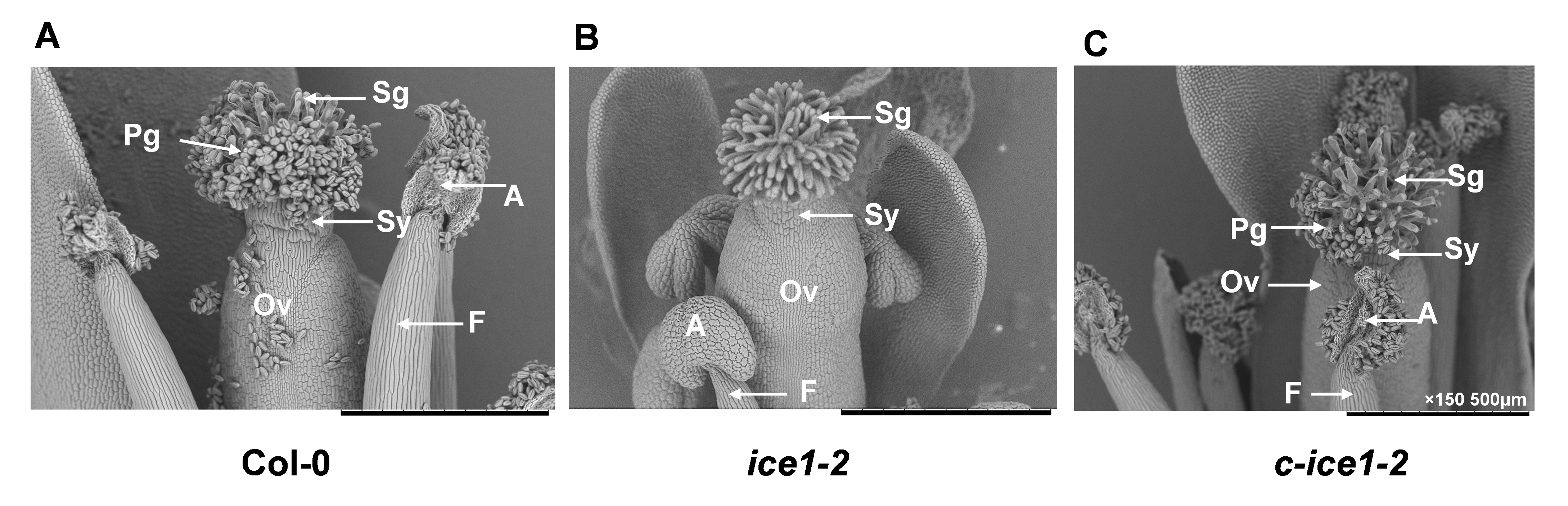

Supplement: S2 Fig — Scanning Electron Microscope (SEM) of flowers from Col-0 (A), ice1-2 (B) and c-ice1-2 (C) at flower stage 14. The pollen grains were released from the dehisced anther locules in Col-0 and c-ice1-2. The ice1-2 pollen grains failed to be released to receptive papillae on the stigma. A, Anther; F, filament; Ov, ovary; Pg, pollen grain; S, sepal; Sg, stigma; Sy, style. (TIF) [file pgen.1007695.s002.tif]

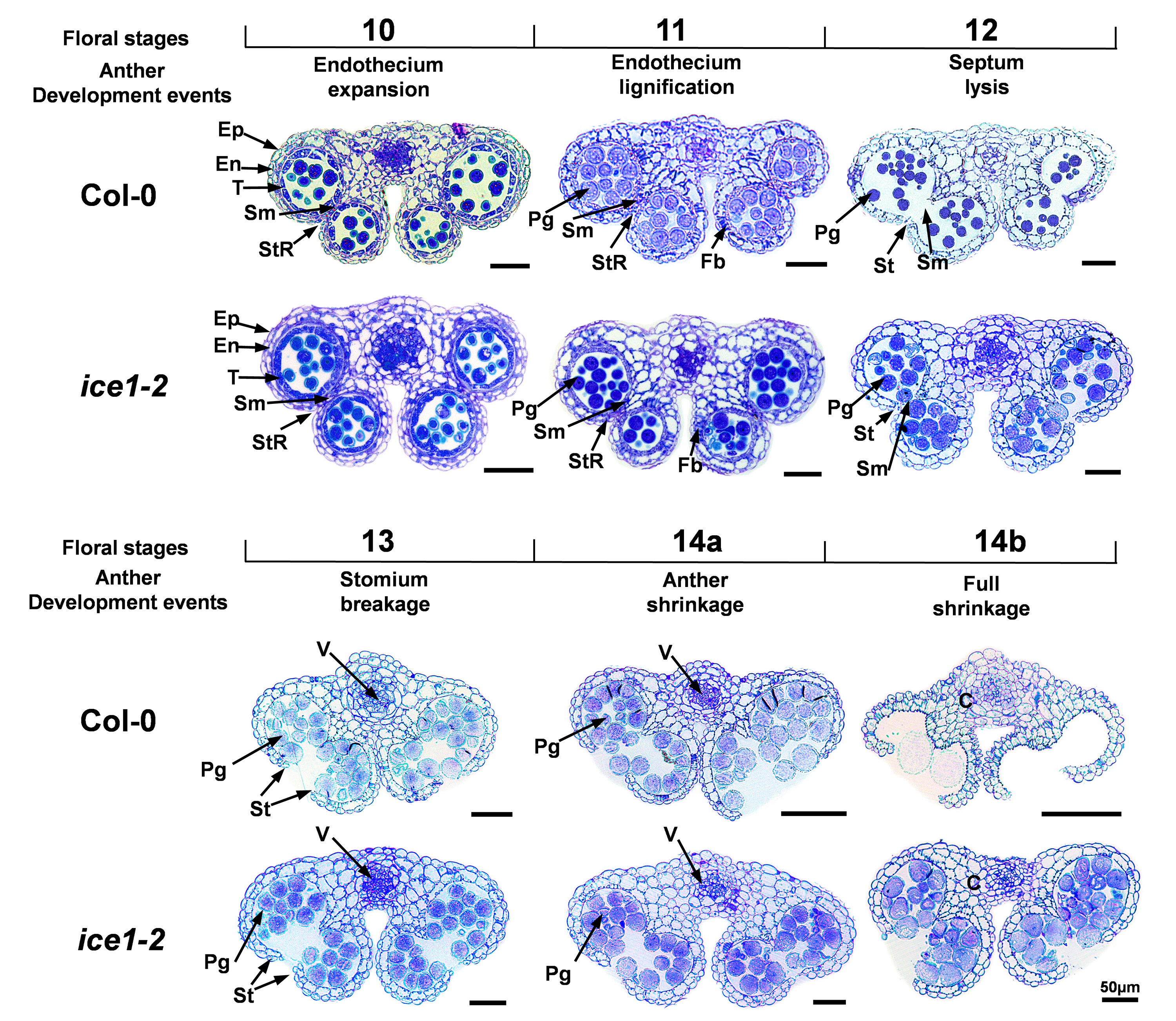

Supplement: S3 Fig — Semi-thin cross sections of anthers from Col-0 and ice1-2 at anther stage 10-14b were stained with toluidine blue. Ep, Epidermis; En, Endothecium; T, Tapetum; StR, stomium region; St, stomium; Sm, septum; Fb, fibrous bands; C, Connective; V, Vascular bundle; Pg, pollen grains. (TIF) [file pgen.1007695.s003.tif]

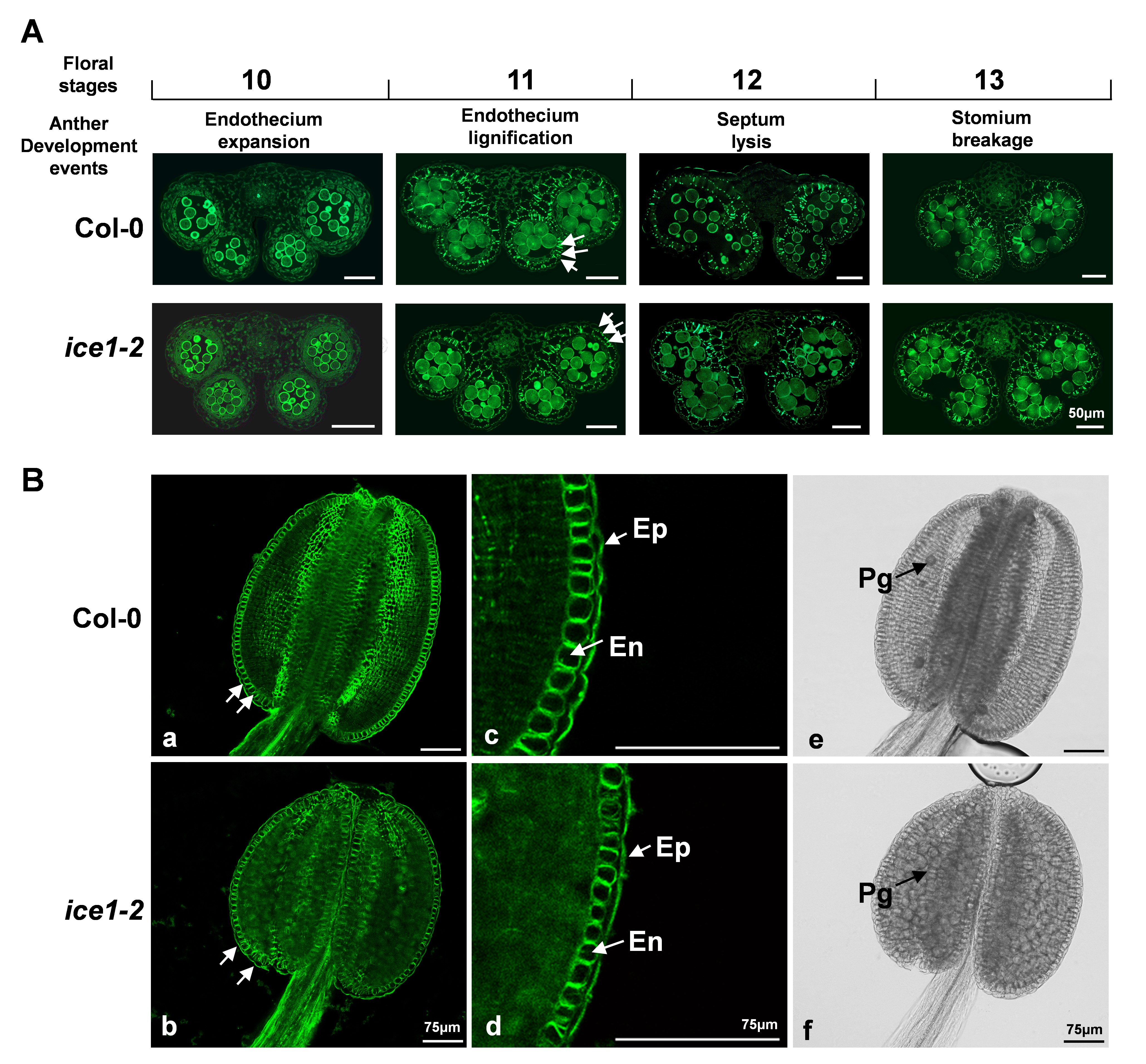

Supplement: S4 Fig — (A) Transverse sectioning of anthers at anther stage 10–13 with auramine O staining. Arrows indicate the positions of endothecium lignification. (B) Fresh anthers at stage 14 with auramine O staining. Secondary thickening is visible in the endothecium (arrows indicated). (a) The anther from Col-0; (b) the anther from ice1-2; (c) Close-up of (a); (d) Close-up of (b); (e) Photographed by bright-field microscopy of (a); (f) Photographed by bright-field microscopy of (b). Ep, Epidermis; En, endothecium; Pg, pollen grains. (TIF) [file pgen.1007695.s004.tif]

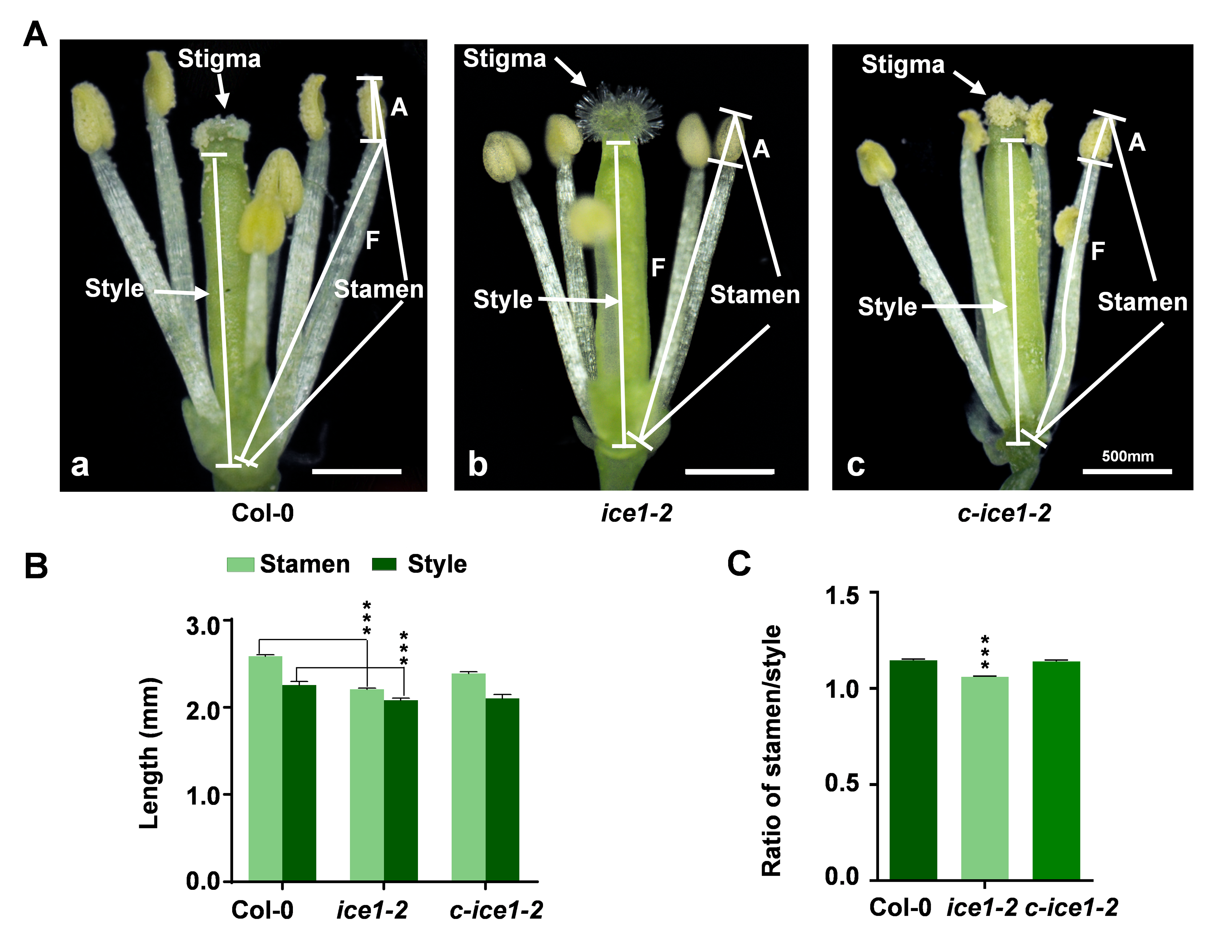

Supplement: S5 Fig — (A) Phenotypes of the stamen and style in Col-0 (a), ice1-2 (b) and c-ice1-2 (c) at flower developmental stage 14. (B) Stamen and style lengths were measured from microscopy pictures (SE, n = 30–39 styles and 119–146 stamens, *** p < 0.001). (C) Ratio of filament/pistil according to length data shown in (B) (SE, n = 119–146, *** p < 0.001). (TIF) [file pgen.1007695.s005.tif]

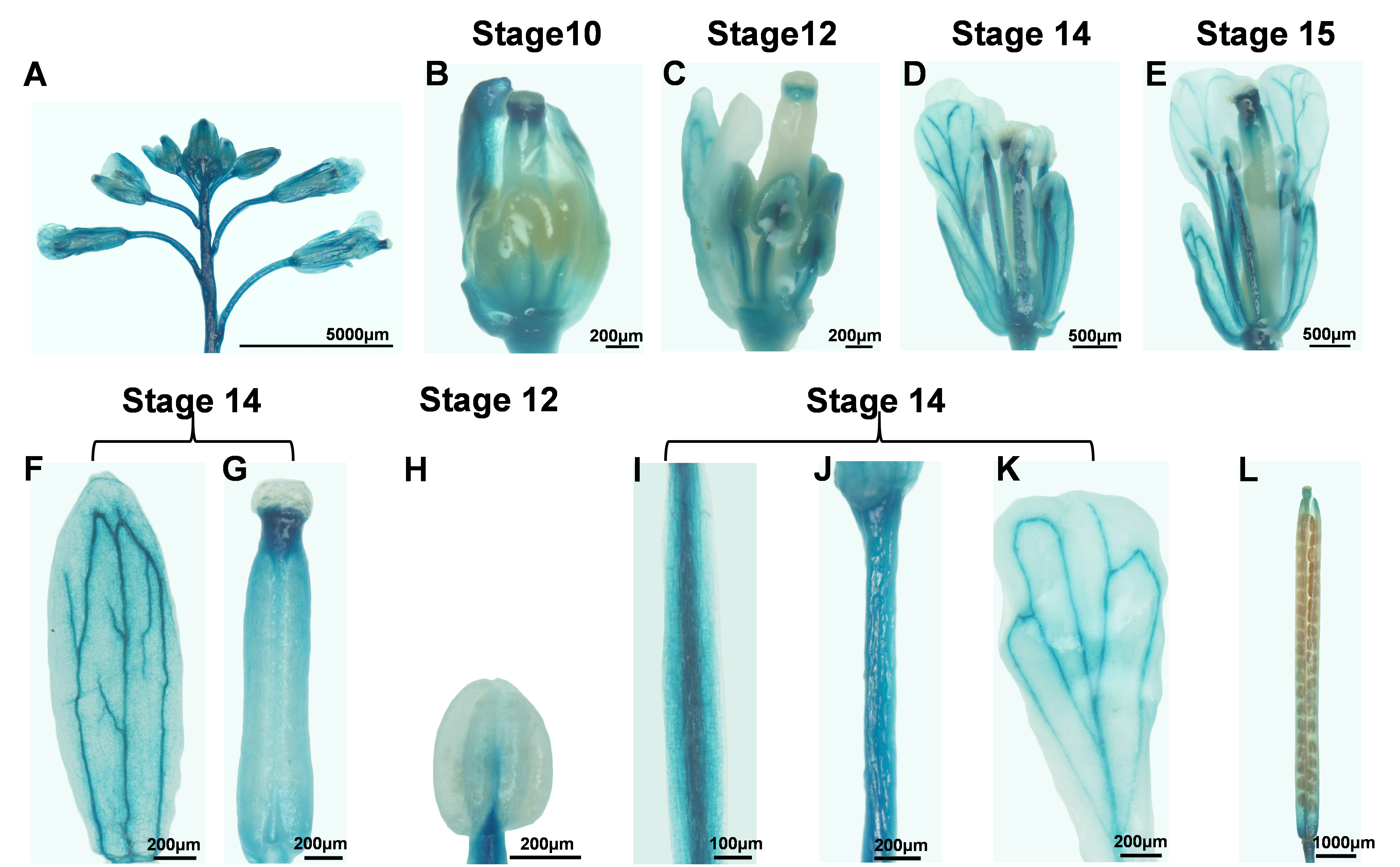

Supplement: S6 Fig — (A) Inflorescence. (B) Flower at flower stage 10. (C) Flower at stage 12. (D) Flower at stage 14. (E) Flower at stage 15. (F) Sepal at stage 14. (G) Pistil at stage 14. (H) Adaxial side of the anther at flower stage 12. (I) Filament at stage 14. (J) Pedicel at stage 14. (K) Petal at stage 14. (L) Silique. (TIF) [file pgen.1007695.s006.tif]

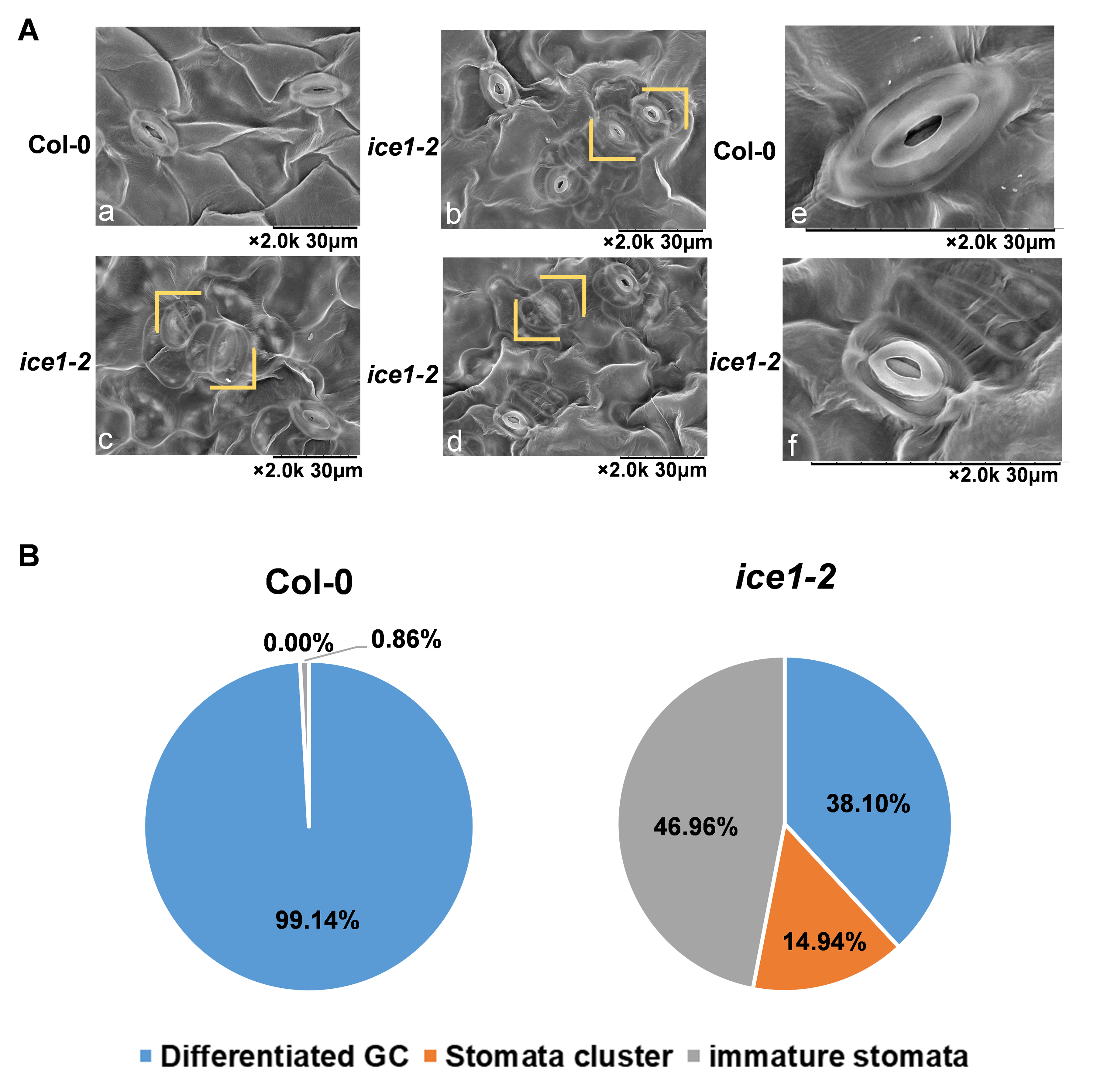

Supplement: S7 Fig — (A) Scanning electron micrographs of stomata from abaxial leaf surface. (a) Mature stomata in Col-0. Yellow brackets show stomatal cluster (b), paired differentiated guard cells (c), and immature stomata (d) in ice1-2. The differentiated guard cells in Col-0 (e) and ice1-2 (f) are also shown. (B) Comparison of proportions of different stomatal types in leaves between Col-0 and ice1-2. (TIF) [file pgen.1007695.s007.tif]

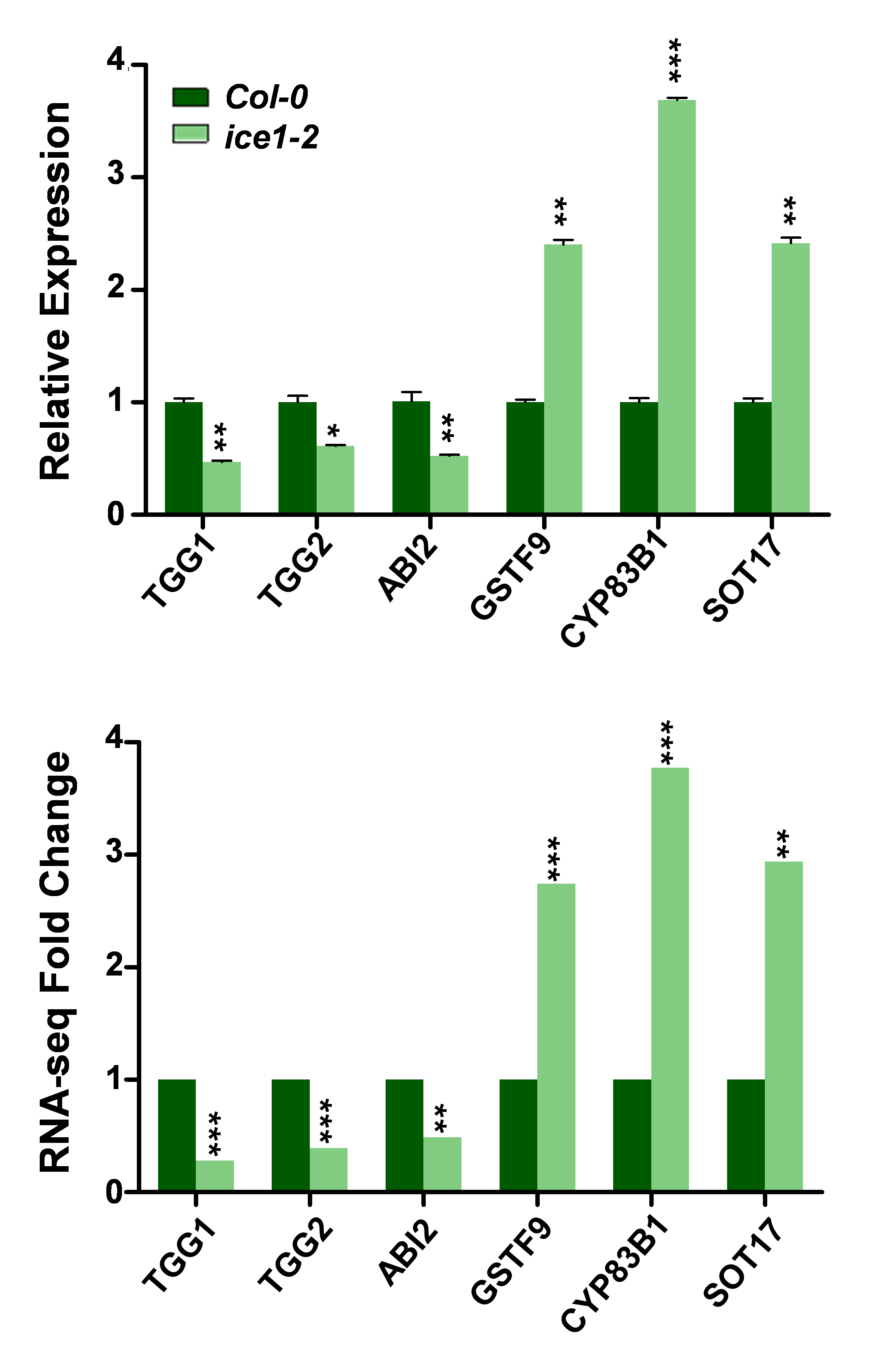

Supplement: S8 Fig — Six genes were selected for comparison of RNA-seq and qRT-PCR results. For RNA-seq data, ** FDR < 0.01, *** FDR < 0.001. For data of qRT-PCR, SE, n = 3, * p < 0.05, ** p < 0.01, *** p < 0.001. Three independent experiments were carried out with similar results. (TIF) [file pgen.1007695.s008.tif]

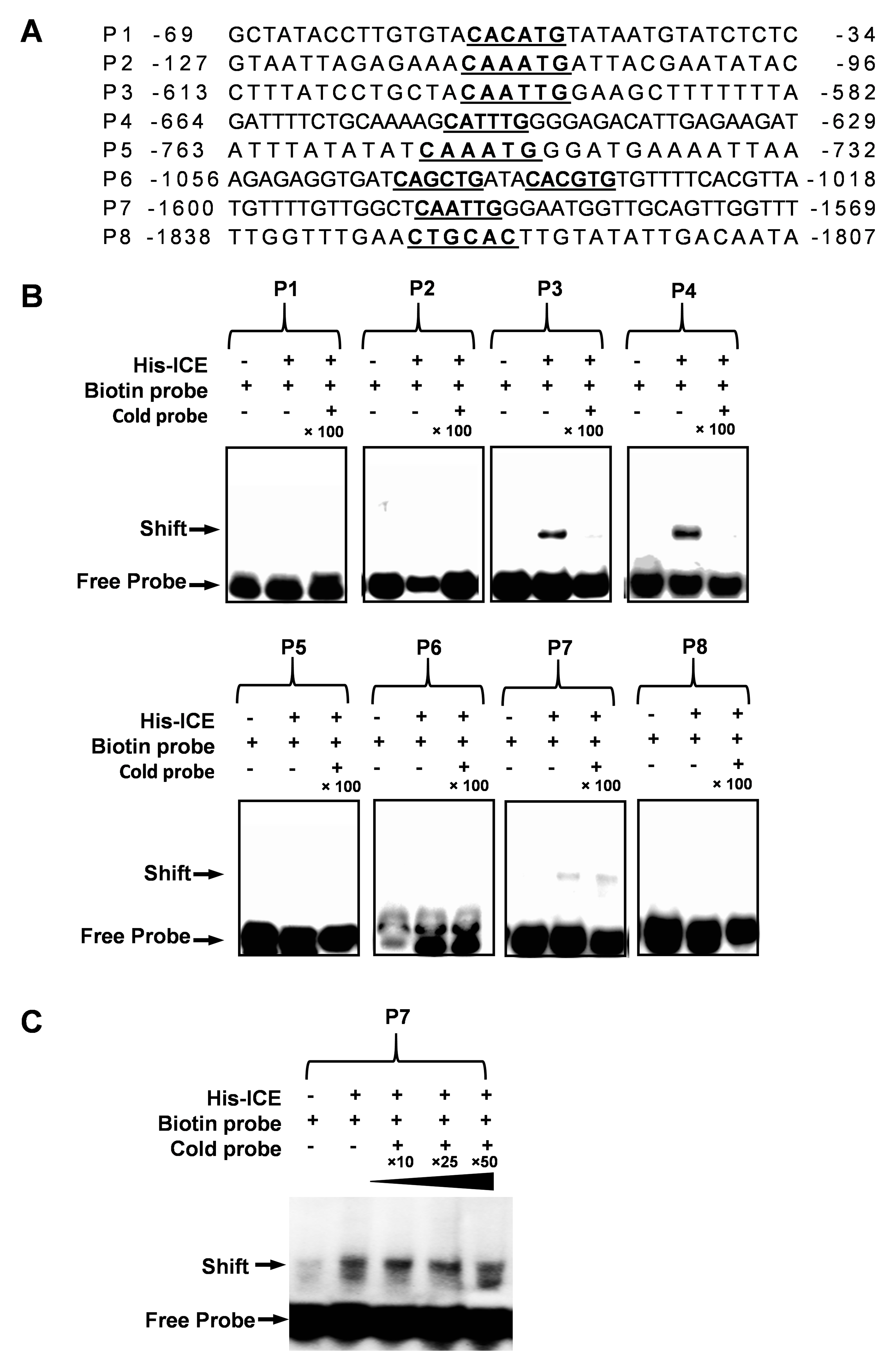

Supplement: S9 Fig — (A) Probe sequences containing nine E-box elements are listed. P6 contains two E-boxes. (B) Binding results of ICE1 to eight probes. P3, P4 and P7 showed binding activity. P3 and P4 exhibited competition by cold probes while P7 did not show competition. (C) P7 did not show competition by cold probes with high concentration. (TIF) [file pgen.1007695.s009.tif]
