# Supplementary material for: INDUCER OF CBF EXPRESSION 1 is a male fertility regulator impacting anther dehydration in Arabidopsis
Source: PLoS Genet. 2018 Oct 4;14(10):e1007695. doi: 10.1371/journal.pgen.1007695 (PMC6191155; doi:10.1371/journal.pgen.1007695)
Supplement: S2 Table — (DOCX) [file pgen.1007695.s011.docx]

**S2 Table. Down-regulated genes that enriched in GO annotations**

| **Accession** | **Gene Name** | **Function** | **Log2FC** | **expression** |
| --- | --- | --- | --- | --- |
| **Hydrolase activity (27)** | | | | |
| AT5G26000 | TGG1/BGLU38 | Myrosinase (Glycoside Hydrolase Family 1)  glucosinolate metabolic pathway | -1.83 | Guard cell, phloem cells of stamen |
| AT5G25980 | TGG2/BGLU37 | Myrosinase (thioglucoside glucohydrolase)  glucosinolate metabolic pathway | -1.35 | Phloem cells of stamen |
| AT5G48375 | TGG3/BGLU39 | thioglucosidase activity  glucosinolate metabolic pathway | -1.35 | Flower |
| AT1G02640 | BXL2 | β-xylosidase (Glycosyl hydrolase family 3)  secondary cell wall metabolism | -1.01 | Flower |
| AT5G20250 | RS6/DIN10 | raffinose synthase 2 (Glycosyl hydrolase family 36)  seed imbibition protein | -1.28 | Mature pollen |
| AT3G57520 | RS2/SIP2 | raffinose synthase 2  seed imbibition protein | -1.21 | Mature pollen |
| AT3G63380 | ACA12 | Ca^2+^ ATPase  Inflorescence growth and seed setting | -1.17 | Guard cell |
| AT3G57330 | ACA11 | Ca^2+^ ATPase  SA-Dependent PCD Pathway | -1.02 | Mature pollen |
| AT1G80660 | AHA9 | H^+^ ATPase  / | -1.18 | Anther |
| AT2G07560 | AHA6 | H^+^ ATPase  / | -1.23 | Mature pollen |
| AT3G52310 | ABCG27 | ABC transporter  / | -1.30 | - |
| At1G26130 | --- | ATPase  male gametophyte development | -1.40 | Mature pollen |
| AT3G10740 | ASD1/ARAF | β-galactosidase (Glycoside hydrolases family 51)  cell wall loosening | -1.28 | Anther |
| AT4G16190 | RD19 | cysteine protease  responsive to dehydration | -1.04 | - |
| AT2G03980 | GDSL-like | GDSL-motif esterase/acyltransferase/lipase  / | -1.06 | Mature pollen |
| AT1G33811 | GDSL-like | GDSL-motif esterase/acyltransferase/lipase  / | -2.65 | - |
| AT2G02970 | APY6 | Apyrase  anther dehiscence and pollen exine pattern formation | -1.10 | Mature pollen |
| AT2G22980 | SCPL13 | serine-type carboxypeptidase  / | -1.04 | - |
| AT1G04600 | XIA | Myosin-like protein  Pollen tube growth | -1.03 | Pollen tube |
| AT3G19960 | ATM1 | Myosin-like protein  / | -1.44 | Stamen |
| AT3G01310 | VIP1/VIH2 | ATP-grasp kinase  Regulates the synthesis of inositol pyrophosphate | -1.05 | Flower |
| AT5G57050 | ABI2 | protein phosphatase 2C  Regulation anion channels of guard cell | -1.03 | Guard cell |
| AT5G02230 | HAD | Haloacid dehalogenase-like hydrolase  / | -1.03 | Mature pollen |
| AT3G44400 | --- | Disease resistance protein  / | -1.09 | Mature pollen |
| AT5G38850 | --- | Disease resistance protein  / | -1.09 | - |
| AT3G62820 | --- | Pectinesterase methylesterase inhibition  Cell wall rigidity | -2.04 | - |
| AT4G16680 | --- | P-loop containing nucleoside triphosphate hydrolases  / | -1.24 | - |
| **Ion transporter (10)** | | | | |
| AT1G11260 | STP1 | Sugar transporter 1  regulate the uptake of sugars | -1.81 | Guard cell of leaf |
| AT3G19930 | STP4 | Sugar transporter 4  responding to environmental stress | -1.13 | Anther and mature pollen |
| AT2G47600 | MHX1 | Na^+^/Ca^2+^ antiporter  cellular metal and/or proton homeostasis | -1.10 | Flower |
| AT3G51860 | CAX3 | cation exchanger 3  regulation of ion homeostasis | -1.11 | Guard Cell |
| AT1G26130 | ALA12 | ATPase  / | -1.40 | Mature pollen |
| AT3G52310 | ABCG27 | ABC transporter  / | -1.30 | - |
| AT4G30110 | HMA2 | ATPase  pollen germination | -1.07 | Anther |
| At4G23700 | CHX17 | Cation/H^+^ exchanger 17  reduced seed set | -1.13 | Anther |
| AT4G01010 | CNGC13 | Cyclic nucleotide gated channel family  / | -1.20 | - |
| AT1G24400 | LHT2 | Amino acid transport  male gametophyte development | -1.02 | Flower |
| **Genes related to water deprivation (5)** | | | | |
| AT5G66400 | RAB18 | dehydrin protein family  ABA and drought response | -5.88 | Guard cells in flower |
| AT3G50970 | LTI30 | dehydrin protein family  positively regulates drought stress | -4.14 | Stamen and guard cell |
| AT4G25480 | DREB1A | AP2 transcription factor  drought stress in the flower | -1.15 | - |
| AT2G18050 | HIS1-3 | Histone 1  drought stress in leaf | -1.12 | Guard cell in leaf |
| AT5G57050 | ABI2 | protein phosphatase 2C  drought stress in leaf | -1.03 | Guard cell |
| **Genes related to auxin response (7)** | | | | |
| AT2G46070 | ATMPK12 | MAP protein kinase  root elongation | -1.43 | Guard cell in stamen |
| AT1G16510 | SAUR41 | Small Auxin Upregulated 41  cell expansion | -1.07 | Vascular leaf |
| AT2G01200 | IAA32 | auxin inducible gene family  male gametophyte development | -1.95 | - |
| AT3G48360 | BT2 | BTB and TAZ Domain Protein  later stages of male gametophyte development | -1.50 | Mature pollen |
| AT4G37610 | BT5 | BTB and TAZ Domain Protein  later stages of male gametophyte development | -1.10 | Mature pollen |
| AT4G27260 | GH3.5/WES1 | gretchen hagen3  Camalexin biosynthesis | -1.03 | Trichome and flower |
| AT5G54510 | GH3.6/DFL1 | gretchen hagen3  / | -1.79 | - |
