# Supplementary material for: INDUCER OF CBF EXPRESSION 1 is a male fertility regulator impacting anther dehydration in Arabidopsis
Source: PLoS Genet. 2018 Oct 4;14(10):e1007695. doi: 10.1371/journal.pgen.1007695 (PMC6191155; doi:10.1371/journal.pgen.1007695)
Supplement: S3 Table — (DOCX) [file pgen.1007695.s012.docx]

**S3 Table. Up-regulated genes that enriched in GO annotations**

| **Accession** | **Gene Name** | **Function** | **Log2FC** | **Expression** |
| --- | --- | --- | --- | --- |
| **JA biosynthesis gene (6)** | | | | |
| AT3G45140 | LOX2 | lipoxygenase 2  JA biosynthesis | 1.24 | Flower |
| AT1G17420 | LOX3 | lipoxygenase 3  anther dehiscent and pollen viability | 1.02 | Flower |
| AT5G42650 | AOS/DDE2 | allene oxide synthase  anther and pollen development | 1.43 | Anther and mature pollen |
| AT3G25780 | AOC3 | allene oxide cyclase 3  JA biosynthesis | 1.14 | Stamen |
| AT2G06050 | OPR3/DDE1 | 12-oxophytodienoate reductase 3  anther dehiscent | 1.54 | Anther |
| AT1G19640 | JMT | jasmonic acid carboxyl methyltransferase  drought responses | 1.14 | Flower and mature pollen |
| **JA responsive gene (14)** | | | | |
| AT3G16470 | JR1/JAC1 | JA-responsive gene  flowering time | 1.27 | - |
| AT3G45140 | LOX2 | lipoxygenase 2  JA biosynthesis | 1.24 | Flower |
| AT5G42650 | AOS/DDE2 | allene oxide synthase  anther and pollen development | 1.43 | Anther and mature pollen |
| AT1G19640 | JMT | jasmonic acid carboxyl methyltransferase  drought responses | 1.14 | Flower and mature pollen |
| AT4G23600 | JR2/COR13 | JA-responsive gene  JA signling | 2.62 | Flower |
| AT3G23250 | MYB15 | MYB family transcription factor  drought responses/lignin biosynthesis | 2.19 | Guard cell of leaf |
| AT1G74430 | MYB95 | MYB family transcription factor  Stamen development | 1.02 | Flower |
| AT5G47220 | ERF2 | AP2 family transcription factor  drought responses | 1.80 | - |
| AT3G52400 | SYP122 | syntaxin protein  pollen tube growth | 1.58 | Mature pollen |
| AT1G28480 | GRX480/ROXY19 | glutaredoxin family  detoxification pathway | 1.29 | - |
| AT5G46050 | PTR3/NRF5.2 | Peptide transporter 3  / | 1.24 | - |
| AT1G49010 | MYBS1 | myb family transcription factor  sugar signaling | 1.19 | Flower |
| AT1G54040 | ESP | epithiospecifier protein  breakdown of glucosinolates | 1.14 | Epidermal cells of sepal |
| AT5G13930 | CHS/TT4 | chalcone synthase  flavonoid biosynthesis | 1.89 | Anther and guard cell of leaf |
| **Flavonoid pathway (8)** | | | | |
| AT5G42800 | DFR/TT3 | dihydrokaempferol 4-reductase  flavonoid biosynthesis | 2.36 | - |
| AT5G13930 | CHS/TT4 | chalcone synthase  flavonoid biosynthesis | 1.89 | Anther and guard cell of leave |
| AT5G05270 | CHIL/TT5 | chalcone-flavanone isomerase  flavonoid biosynthesis | 1.84 | - |
| AT3G51240 | F3H/TT6 | flavanone 3-hydroxylase  flavonoid biosynthesis | 2.07 | Flower |
| AT5G07990 | F3’H/TT7 | flavonoid 3-hydroxylase like  flavonoid biosynthesis | 1.01 | - |
| AT5G63580 | FLS2 | flavonol synthase  flavonoid biosynthesis | 1.82 | Flower |
| AT1G06000 | UGT89C1 | flavonol-7-O-rhamnosyltransferase  flavonoid biosynthesis | 2.02 | - |
| AT1G30530 | UGT78D1 | flavonol 3-O-rhamnosyltransferase  flavonoid biosynthesis | 1.22 | - |
